# Overuse of medical imaging and effects of payer-provider integration: quasi-experimental evidence from Finland

**DOI:** 10.1186/s13561-025-00592-0

**Published:** 2025-01-28

**Authors:** Konsta Lavaste

**Affiliations:** 1 Finnish Institute for Health and Welfare, PO Box 30, 00271 Helsinki, Finland; 2 University of Jyväskylä, PO Box 35, 40014 Jyväskylä, Finland

**Keywords:** Medical imaging, Medical overuse, Payer-provider integration, Financial incentives, Finland

## Abstract

**Background:**

Healthcare expenditures have risen in middle- and high-income countries. One of the potential contributors is the overuse of diagnostics. I explore whether medical imaging is overused when privately owned clinics in Finland treat patients with voluntary private health insurance (VPHI).

**Methods:**

I employ administrative insurance claims data from a major Finnish insurance company, covering 2016–2019, and exploit two market entries of clinics owned by the company in 2017. The underlying assumption is that the insurance company’s own clinics had weaker incentives to overuse imaging than other privately owned clinics because the payer and the provider belonged to the same entity. I identify the overuse using the staggered difference-in-differences (DID) strategy, in which I consider patients from cities with a market entry as the treatment group and compare them to patients in other similar cities.

**Results:**

I find that the market entries decreased the use of radiography and ultrasound imaging in the treatment of VPHI policyholders, suggesting that private clinics overused these imaging technologies. The more expensive computed tomography (CT) and magnetic resonance imaging (MRI) were, however, not overused.

**Conclusions:**

The results show that private clinics in Finland overused some imaging technologies when treating VPHI policyholders. The extent and magnitude of overuse can, however, vary considerably between imaging technologies and medical ailments.

**Supplementary Information:**

The online version contains supplementary material available at 10.1186/s13561-025-00592-0.

## Background

Healthcare expenditures have risen drastically in middle- and high-income countries in recent decades. In 2000–2019, the average ratio of health expenditures to gross domestic product in European Union member states rose from 6.9% to 8.3% and from 12.5% to as high as 16.7% in the United States [1]. The trend was similar in Finland: the ratio rose from 7.1% to 9.2%. The problem is that the increases in expenditures have led to much smaller (or nonexistent) improvements in health outcomes, such as mortality or life expectancy [2, 3]. One plausible contributor to the disparity is the overuse of healthcare services (such as diagnostics), which means the provision of services in which expected costs are higher than expected benefits. This overuse increases expenditures but less often improves health outcomes. The costs of such low-value imaging are indeed estimated to be high [4].

This study aims to detect whether financial incentives have led to overuse of medical imaging. Unlike the scarce existing research on the topic, I use an identification strategy that exploits market entries of clinics owned by an insurance company and compares differential effects among imaging technologies. I focus the analysis on the treatment of patients who had voluntary private health insurance (VPHI) which allows preferential access to private clinics for people who do not want to use universal public healthcare services in a system similar to the National Health Service (NHS) in the United Kingdom [5].

The overuse of diagnostics has attracted increasing attention from researchers and policymakers in recent decades [6–10]. Such overuse can be caused by various factors, but financial incentives are likely contributors because physicians and providers respond to them [11–14]. Health insurance can amplify the overuse if an insured patient’s marginal costs are low (the level depends on cost-sharing) because, from the patient’s point of view, it makes services with low expected benefits worthwhile.^1^

Overuse is difficult to analyse because the benefits and harms of a service are not easy to quantify, and the appropriateness of a service varies between patients [6]. Rather than determining overuse separately for each patient, I circumvent the quantification problem by identifying overuse by comparing imaging tendencies in clinics that had financial incentives for overuse to clinics that did not. More precisely, the empirical strategy exploits the market entries of private clinics owned by the insurance company.

The insurance company’s ownership of a clinic is a prime example of payer-provider integration, which is used in referring to a close partnership between the payer and the provider.^2^ The point of payer-provider integration is that it aligns incentives of the payer and the provider [17]. The integration implies that incentives for excessive care were weaker in the insurance company’s clinics than in other private clinics if the patient had a VPHI from the same insurance company that owned the clinics. It was in the interest of the insurance company’s clinics to avoid excessive services for the patients it had insured because the company itself had to pay the associated expenses. By contrast, in all the other private clinics, the provider (i.e. clinic) and the payer (i.e. insurance company) were different entities with a conflict of interest: profit-driven providers were reimbursed on a fee-for-service basis and, hence, had an incentive to maximise service provision.

Meanwhile, the imaging probability of an insured patient should not have depended on the clinic (all else being equal) because the nationally set Current Care Guidelines apply equally to all healthcare providers and professionals [18]. Therefore, the possible differences in clinics’ imaging tendencies should result from (i) differences in providers’ incentives and (ii) non-random patient selection into clinics.^3^ In order to rule out selection, I take the market entries of insurance company’s clinics as city-wide shocks on VPHI policyholders’ treatment: after the entries, a majority of policyholders’ physician visits directed to the insurance company’s clinic and, hence, were subject to different financial incentives than before. Thus, I can compare the study company’s VPHI policyholders in the market entry cities (treatment group) to policyholders in other major cities (control group) regardless of the clinic they attended. Furthermore, if systematic inter-city differences in patient case-mix and healthcare market conditions are controlled, comparisons between the treatment and control group before and after the market entries identify the possible overuse (or underuse).

### Literature

My analysis of healthcare service overuse concentrates on imaging services because they are a potential vehicle of such overuse as their availability and use have increased across OECD countries [19], and there is a large body of medical literature studying their overuse. Imaging underuse is also a potential problem, but it is much less frequently observed in the literature than overuse [8]. In many cases more than 30 percent of imaging is considered to be of low-value [9].^4^ According to literature reviews [7–10], magnetic resonance imaging (MRI) is overused for knee and lower back pain, whereas computed tomography (CT) is overused for minor head injuries, to name a few examples. The overuse is often attributed to healthcare professionals’ (i) insufficient knowledge on the right use of imaging, (ii) reliance on established clinical practice, (iii) fear of malpractice, and (iv) financial incentives; as well as (v) patients’ expectations toward more advanced diagnostics [9]. Moreover, the overuse is not limited to private healthcare. The medical literature includes evidence of imaging overuse in public healthcare systems, such as England’s NHS [20, 21] and Finland’s public hospitals [22, 23].

The economic literature focuses on the financial mechanisms behind physicians’ treatment decisions and healthcare overuse. Competitive pressures incentivise providers to generate income through overuse; thus, the consistently observed positive correlation between physician density and healthcare consumption has been interpreted as an indicator of provider overprovision—often labelled as physician-induced demand [24]. In addition, changes and differences in remuneration systems have revealed that increases (decreases) in physicians’ financial incentives lead to greater (lesser) provision of care, showing that either underprovision or overprovision exists [13, 15, 25–27]. Gottschalk et al. [28] have confirmed physician-induced overprovision in a field study,^5^ and at least four papers have studied physicians’ financial incentives and overuse specifically in imaging services. Exploiting a reimbursement reform in Switzerland, Zabrodina et al. [29] showed that hospitals overutilised CT and MRI through repeat examinations, that is, extra examinations on top of the initial examination. Ikegami et al. [30] found that Japanese hospitals compensated for the loss of patients by increasing MRI scans per patient. Chalkley and Listl [31] and Kalmus et al. [32] demonstrated that dentists who were reimbursed through a fee-for-service model were more likely to assign small dental radiographs (dental films) than salaried dentists.

### Contribution

I provide new insights to the economic literature by studying the overuse of imaging through an empirical strategy that exploits payer-provider integration rather than differences in remuneration systems or the competitive environment. Also, only a few papers have investigated imaging services, despite the fact that excess imaging (unlike the overuse of many other treatments) is not just wasteful but also potentially harmful to the patient’s health due to ionising radiation [31]. Another advantage of this paper is that it separates the use of different imaging technologies. Some technologies might have been overused while others have been underused. Moreover, I consolidate the medical side of the overuse literature, which concentrates heavily on North America and Australia [7, 10].

I also contribute to the literature concerning the effects of payer-provider integration on physicians’ treatment choices and cost-containment. Earlier studies in the United States have found that the practices of managed care (a form of payer-provider integration) contained costs, while later research does not support this finding [33]. European studies are less numerous, mixed in their results (with some finding cost-containment effects and others not), and some are possibly biased by self-selection of individuals into insurance plans [33–37]. In addition to analysing cost-containment, I study the effects on physicians’ treatment choices which have, to my knowledge, been explored less. The few studies that exist have found that payer-provider integration decreases probabilities of surgery [36] and physiotherapy [37]. I also contribute by studying a setting in which the integration is ownership-based rather than contract-based.^6^ It is not evident that a contract removes the conflict of interest between the payer and the provider [17], which may dilute the cost-containment. In my setting, however, the insurance company’s clinics were owned and operated (thus heavily steered) by the company.^7^ Lastly, I contribute by studying the effects of payer-provider integration when patients’ choice of provider is not restricted—or even incentivised by differing reimbursement rates—to selected providers. This implies that the effects of payer-provider integration might be milder here than in other settings because patients with strong preferences toward certain care practices (such as abundant use of imaging), might choose to attend other private clinics.

### Market entries of clinics owned by an insurance company

The Finnish healthcare system provides services through three distinct systems: public, occupational and private healthcare [18]. It is typical that people use services from multiple systems; in 2013, for example, approximately half of the population did so [38]. First, the tax-funded public healthcare system (similar to the NHS in the United Kingdom) provides services universally through health centres and hospitals with relatively low patient fees,^8^ although waiting times tend to be long and specialists cannot be accessed without a general practitioner’s (GP) referral [18]. Second, employers often purchase occupational healthcare coverage for their employees, who can then access occupational healthcare services (provided by private clinics) with short waiting times and without patient fees [18]. Third, private healthcare services are the only alternative to public healthcare for people not entitled to occupational services. Private services are relatively expensive, but patients can access them with minimal waiting time, and a visit to a specialist does not require a GP’s referral [18]. Geographical availability of private healthcare is, however, limited because Finland is a sparsely populated country and more than half of the municipalities do not have any private clinics (see Supplementary Appendix A).

This study concentrates on service use within the private healthcare system and, more specifically, patients with a voluntary private health insurance (VPHI). In the private healthcare system, the statutory National Health Insurance reimburses a small part of costs,^9^ and the remainder must be covered through a VPHI or out-of-pocket. Hence, the VPHI market has developed to serve people who prefer to use private healthcare services but do not wish to cover the relatively high costs out-of-pocket. The VPHI policies typically have a (self-selected) annual deductible of 100–1,000 euros and all the costs exceeding this are reimbursed without copayments. The majority of the policies are self-purchased (rather than group policies) and cover healthcare expenses incurred due to accidents and/or illnesses [5, 41].

The study company is the market leader in VPHIs in Finland, with an approximately 40% market share in 2016–2018 [42]. From 2013 onward, it expanded from financing to the provision of healthcare services by establishing its own private clinics (see Supplementary Appendix B for a map). The cities chosen were among the ones with the most extensive supply of healthcare services and the largest population.^10^ Initially, the main intention was to enhance and shorten treatment spells, which would subsequently translate into higher quality and expenditure savings by reducing sick leave compensations [36].^11^

The company made great efforts to align its physicians’ behaviour with the company’s objectives of cost-containment. For example, diagnostic criteria were standardised and recent research on unnecessary knee and shoulder surgeries was employed to educate physicians on a weekly basis [36]. Also, recruitment of physicians to the clinics was based on physicians’ attitudes toward the clinics’ goals and business idea [36]. Hence, I expect that physicians had considerably lower incentives to provide unnecessary services than in other private clinics.

During the study period, the study company’s clinics were open to everyone, although most of the patients had an insurance policy from the company. The policyholders were not mandated to use the company’s clinics; instead, they were free to choose any private clinic in Finland without an effect on cost-sharing (which consisted only of an annual deductible), although there might have been other costs, such as travelling expenses. For these policyholders, the benefit of choosing one of the insurance company’s clinics was to avoid most of the paperwork and wait time associated with the processing of payment commitments (in addition to possible benefits regarding travel distance and expected care quality).

The study company reimbursed all clinics on a fee-for-service basis. Normal private clinics had an incentive to maximise service provision for the company’s VPHI policyholders: each additional service provided more income. By contrast, the insurance company’s clinics had weaker incentives to overuse services when treating policyholders of the company because the company itself had to pay the associated costs. A purely economical analysis would suggest that clinics of a profit-maximising insurance company even had incentives to underuse services for its own policyholders, but that would have been limited by (i) physicians’ imperfectly aligned incentives and (ii) patients’ freedom of choice. More precisely, the company reimbursed physicians in its clinics through fee-for-service (rather than on a salaried basis) which means that if the physicians underused services in study company’s clinics, the underuse would have come at the expense of both their personal income and patients’ health (assuming that underuse of imaging leads to inappropriate treatment decisions). Also, if the study company’s clinics had underused services and provided more inappropriate treatments, there probably would have been reputational effects and insured patients—who were free to choose any private clinic in Finland without an effect on cost-sharing—might have begun avoiding the company’s clinics. Such avoidance was not observed in the data: in 2018, approximately 70% (40%) of policyholders visited study company’s own clinics when seeking care for an accident (an illness) (see Fig. 3).

### Medical imaging and its overuse

Imaging is a valuable tool in physicians’ clinical decision making. Each time the physician needs to decide whether to use imaging and, if there are more than one viable technology, which one to use. These choices are shaped by demand (e.g. price and patient’s preferences), supply (e.g. financial incentives) and situational factors (e.g. local availability of imaging equipment) [14].

**Fig. 1:**
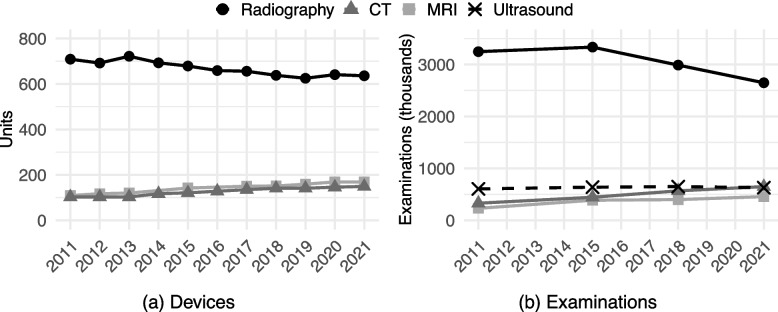
Number of selected imaging devices and examinations in clinical use in Finland. *Notes:* The values concern combined numbers of devices and examinations in all healthcare sectors (public, private, and occupational). Radiography excludes dental devices and examinations. The number of MRI devices in 2014 and 2016 are unavailable. The number of ultrasound devices is also unavailable. Data on radiography and CT devices are from Radiation and Nuclear Safety Authority [44–54]. Data on MRI devices were received from the Radiation and Nuclear Safety Authority by email. Data on the number of examinations are from Ruonala et al. [55]. The number of MRI and ultrasound examinations are subject to greater survey bias than radiography and CT examinations [56]

Radiography (i.e. plain X-ray) and ultrasound are less expensive technologies that expose patients to small (or nonexistent) amounts of ionising radiation. The images they produce are, however, not very detailed. In contrast, magnetic resonance imaging (MRI) and computed tomography (CT) are more expensive but provide a very detailed picture. Their downsides are that MRI is relevant only for some tissues (e.g. joints and the central nervous system) and CT exposes the patient to a considerable radiation dose. Figure 1 shows that in 2016–2018, radiography was by far the most common technology by the number of devices (a) and examinations (b). Conversely, MRI and CT devices were not plentiful but became more common over time [43].

Imaging should not, however, be applied carelessly. Besides causing expenses, it may also inflict mental and/or physical harm to the patient [6]; hence, if applied when unnecessary, its benefits are not likely to exceed the costs. I refer to this unnecessary use of imaging as “imaging overuse” (following Brownlee et al. [6]). Conversely, it is possible that imaging is not applied even if it would be necessary, leading to “imaging underuse”.

Because the assessment of costs and benefits of imaging requires advanced knowledge of medical science, patients must rely on physicians’ decisions. This information asymmetry enables physicians to overuse imaging by belittling costs or exaggerating benefits. In practice, there are multiple ways in which providers can overuse imaging. The physician can choose to employ imaging without expecting to find anything (e.g. “just in case”). Also, the physician can employ more expensive imaging technology than is necessary (e.g. a CT scan instead of a radiograph), which is problematic when the cheaper alternative is equally effective and exposes the patient to a smaller amount of radiation. Physician and/or patient risk aversion can also contribute to the excessive use of more advanced technologies [14]. In addition, patients with strong preferences may demand certain services from their physicians.

Nationally set healthcare guidelines try to tackle imaging overuse by prohibiting low-benefit imaging in, for example, nonspecific acute lower back and neck pain [57, 58]. The overuse of imaging is, however, recognised as an issue in Finland because the Radiation and Nuclear Safety Authority [59] guidelines explicitly discouraged physicians from writing imaging referrals just to appease their patients.

It is important to note that capacity constraints may affect clinics’ ability to overuse imaging. Hence, some local private healthcare markets may not have had capacity to overutilise MRI and CT, which were supplied by considerably fewer units than radiography. This speculation is supported by extreme variations in imaging prices across cities and firms. For example, in 2018, one of the largest private clinic chains had set the price of an extensive MRI examination at 599 euros in one city and 259 euros in another [40]. Moreover, a recent market entrant was able to provide MRI examination for 364 euros, while an incumbent clinic provided the same service in the same city for 495 euros [60, 61]. Because each of the study company’s clinics was equipped with radiography, ultrasound, MRI and CT devices, it is a plausible alternative hypothesis that the market entries of its clinics had a significant positive impact on imaging capacity in the respective cities. Thus, it is possible that the market entries facilitated a reduction of imaging underuse (through increased imaging capacity) rather than overuse (through payer-provider integration) in the case of MRI and CT imaging.

## Methods

### Data

*Data.* I use nationwide administrative claims data from the study company. The data cover all three million claims on the study company’s self-purchased non-statutory health insurance policies paid in 2016–2019. Each claim contains information on the service type^12^ and price of the service. In order to create comparable observations, I (i) exclude claims paid after one year from the accident or the onset of illness,^13^ (ii) aggregate the data from the claim level to the accident-/illness-level and (iii) restrict the data to accidents/illnesses that took place in 2016–2018. This results in a dataset of reimbursed accidents and illnesses with an equally long (one-year) follow-up period. I further exclude accidents/illnesses for which reimbursement was only for medicines or physiotherapy because in these cases, the probability of imaging was zero. See Supplementary Appendix D for more information on the data.

*Sample selection.* I separate accidents and illnesses into different samples. In addition, I construct alternative samples for four conditions commonly studied for imaging overuse: traumatic head injury, non-traumatic lower back pain, non-traumatic knee pain and non-traumatic neck pain. I restrict each of the samples to working-age adults (18–64 years old) and persons living in a specific set of treatment and control cities. The two cities with a market entry in 2017 form the treatment group. The rest of the Finnish municipalities (only largest of which are called cities) are heterogeneous in their private healthcare markets (including imaging capacity) and sociodemographic structures (see Supplementary Appendix A); hence, the control group is necessarily suboptimal. I decided to include all cities with at least 60,000 inhabitants in 2017 in the baseline control group (excluding cities with one of the study company’s clinics and those in the capital region) and test the robustness of the results with alternative control groups. All of the baseline control cities (${N=10}$) are sufficiently distant from treatment cities in order to discourage patients in control cities from seeking care in treatment cities (see map in Supplementary Appendix B). The final baseline samples include 8, 496 accidents and 29, 332 illnesses.

*Outcomes.* The main outcome is a binary indicator that equals 1 whenever the monetary sum of imaging claims under the same accident/illness identifier is greater than zero. The binary outcomes measure the extensive margin of imaging use. Because I want to compare imaging overuse between technologies, I create separate outcomes for them. Unfortunately, the claims data bundle radiography reimbursements with ultrasound reimbursements, as well as MRI reimbursements with CT reimbursements. Thus, I construct three outcomes: binary indicators for the patient undergoing (i) radiography or ultrasound, (ii) MRI or CT and (iii) any imaging within one year from the accident or the onset of illness. To analyse cost-containment, I also construct the monetary sums of claims concerning (iv) radiography and ultrasound, (v) MRI and CT, (vi) all imaging and (vii) all (imaging and non-imaging) expenditures within one year of the accident or the onset of illness. The measure of all expenditures expands the analysis also beyond imaging.

*Other variables.* The claims data include the city of residence, sex and age of the patient at the time of the accident or the onset of illness. I construct indicators of age bins with 10-year bands. I also control for the type of injury or illness (e.g. injury by falling or knee-region illness) and the injured body part (e.g. wrist), both of which are readily available in the data. In the robustness analyses, I also control for insurance policy characteristics, namely the level of cost-sharing and insurance product fixed effects.

### Empirical approach

In order to rule out selection into clinics, I exploit the fact that the study company opened clinics in two cities in 2016—in the middle of the observation period. I identify overuse by comparing imaging tendencies among study company’s VPHI policyholders in the cities with a market entry (${N=2}$) to selected cities without such market entry (${N=10}$), without taking into account which type of clinic actually provided the care in each case. More precisely, I employ a difference-in-difference (DID) strategy with staggered treatment adoption because the entries were not simultaneous. This method might, however, provide biased estimates if there were systematic differences in patients’ characteristics between the cities. For example, patients in market entry cities might undergo more imaging because they face more serious illnesses or accidents, are on average older, or possess policies with lower cost-sharing. Hence, I control for patient characteristics, municipality fixed effects, as well as accident and illness type in all the estimations, and the level of cost-sharing in the robustness tests.^14^

I estimate the staggered DID estimates using an event study specification because it reveals dynamic treatment effects and enables analysis of the parallel trends assumption (see Miller [62] for more information). The estimation equation is:

1 $$\begin{aligned} y_{imt} = \alpha _0 + \sum \limits _{a=-6}^{+6} \alpha _{1,a} \mathbbm{1}[t-t^{\text {entry}}_{m}=a] \times \text {Treated}_{m} + \mathbf {\alpha }_2 \textbf{X}_{it}' + \lambda _{t} + \mu _{m} + \varepsilon _{imt}, \end{aligned}$$

where $y_{imt}$ is the outcome for patient *i* from city *m* in time (year-quarter) *t*. ${\text {Treated}_{m}}$ is the treatment variable, which equals 1 if the patient lived in one of the treatment cities at the time of the accident or the onset of illness. I construct a set of time-to-entry indicators (${\sum _{a=-6}^{+6} \mathbbm{1}[t-t^{\text {entry}}_{m}=a]}$) which equal 1 if *t* minus city-specific market entry timing (${t^{\text {entry}}_{m}}$) equals the time-to-entry index (*a*). There are six pre-entry periods and six post-entry periods, in addition to the entry period. I use a standard practice in which the period before entry is the baseline (${\alpha _{1,-1}=0}$).

$\textbf{X}_{it}$ is a set of controls that includes age and sex indicators as well as fixed effects for accident or illness type. The controls take into account inter-city differences in the patient case-mix. I do not control for the insurance policies’ characteristics in the baseline estimations in order to avoid controls which are potentially affected by the market entries. $\lambda _{t}$ includes the time fixed effects that capture changes in national imaging trends over time. $\mu _{m}$ are the city fixed effects, which take into account regional differences in popularity of imaging (as documented, for example, by Berger and Czypionka [63] in Austria) as well as the regional availability and prices of imaging services. ${\text {Treated}_{m}}$ is absorbed by $\mu _{m}$ and time-to-entry indicators by $\lambda _{t}$. The error term $\varepsilon _{imt}$ includes the unobserved characteristics. Standard errors are clustered at the city level (${N=12}$), because (i) the treatment is assigned at the city level and (ii) Finland has regional differences in population health [64]. Hence, the imaging prevalence is likely to be correlated between patients from the same city.

Interactions of the time-to-entry indicators and the treatment variable (${\text {Treated}_{m}}$) estimate the coefficients of interest ${\sum _{a=-6}^{+6} \alpha _{1,a}}$ which are presented visually in the results. The choice of the estimator is not straightforward as the performance of the traditionally used two-way fixed effect (TWFE) estimator has been recently found to be weaker in staggered designs, but the properties and relative performance of the new estimators (of which there are plenty) are rather ambiguous. The strength of the newer methods is that they take treatment effect heterogeneity into account, but since my setting has only two treated groups, the potential for heterogeneity is limited. Nevertheless, I estimate the results using both (i) TWFE estimator and (ii) Callaway and Sant’Anna [65] estimator, which is a common choice for allowing treatment effect heterogeneity across treated cities [66].^15^ If the post-entry coefficients (from $\alpha _{1,0}$ to $\alpha _{1,+6}$) are negative and statistically significant (and the pre-entry estimates from $\alpha _{1,-6}$ to $\alpha _{1,-2}$ are not different from zero), it can be concluded that the market entries decreased the imaging tendency and, thus, private clinics overused imaging. Conversely, positive and statistically significant post-entry estimates imply imaging underuse.

The estimation strategy has two main weaknesses. First, the choice of control group is not evident because the private healthcare markets vary considerably across the country. The baseline analysis uses the ten largest cities as the control group, even if a smaller or larger number would also be justifiable. Second, the staggered DID strategy requires that the pre-treatment outcome trends are parallel between treatment and control groups. The fulfilment of this assumption is always debatable and difficult to show with certainty. A potential way to overcome these weaknesses is to use an alternative estimation strategy: the generalised synthetic control method (GSCM). GSCM constructs a synthetic control group by calculating optimal weights for control units based on the outcome and the covariates. The method allows for multiple treated units and staggered treatment timing, without requiring parallel pre-treatment trends requirement or an arbitrary choice of a control group. The weakness of the method is, however, that it requires time series data, whereas each accident/illness is observed only once. Hence, the data must be aggregated to the area level to obtain a time series. The aggregation makes the data and estimation less precise; hence, I employ DID as the benchmark method and conduct GSCM estimations only as a robustness test. See more discussion on the control group selection in Supplementary Appendix A.

## Results

### Descriptive statistics

Table 1 shows descriptive statistics of the outcome variables in the main estimation samples. Fourteen percent of the policyholders’ accidents were reimbursed for radiography or ultrasound, 24% for MRI or CT and 36% for any imaging (panel A). The two-percentage-point difference results from accidents that were reimbursed for multiple modes of imaging. Imaging was less common among the treatment of policyholders’ illnesses: 10% underwent radiography or ultrasound, 7% MRI or CT and 16% underwent any imaging (panel B).

Supplementary Appendix F Table A1 shows that 18–37-year-olds are more common in the estimation samples than older persons. In addition, half of the reimbursed accidents and 72% of the reimbursed illnesses concerned females. Depending on the sample, 63%–73% of the patients lived in the control cities and the rest in the two market entry cities. Supplementary Appendix G Figure A1 shows that the number of reimbursed accidents increased over time in one market entry city while remaining relatively stable in the second market entry city and the control cities. The number of reimbursed illnesses increased in treatment and control cities.

**Table 1:** Summary statistics of the main outcome variables

	Mean	SD	Min	Max	N
*Panel A. Accidents*					
Radiography or ultrasound	0.14	0.34	0	1	8,496
MRI or CT	0.24	0.43	0	1	8,496
Any imaging	0.36	0.48	0	1	8,496
*Panel B. Illnesses*					
Radiography or ultrasound	0.10	0.30	0	1	29,332
MRI or CT	0.07	0.25	0	1	29,332
Any imaging	0.16	0.37	0	1	29,332

Includes accident-/illness-level observations in 2016–2018. Outcomes are based on claims within one year from the accident or the onset of illness

**Fig. 2:**
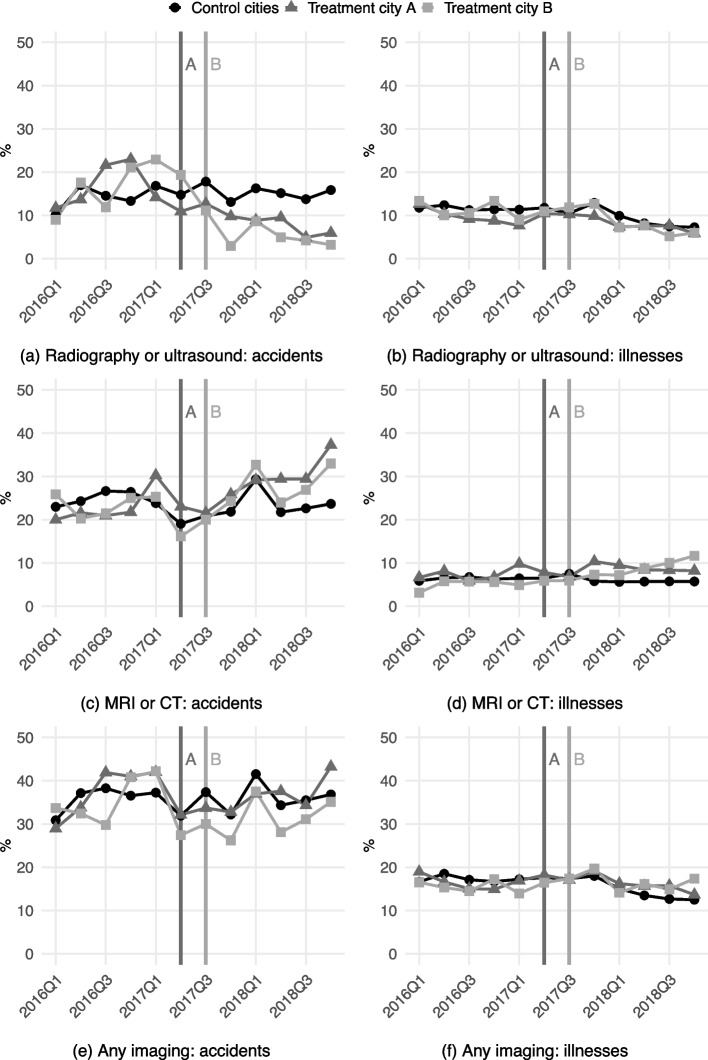
Outcome means. *Notes:* Based on accident-/illness-level data. The means were calculated by dividing the number of policyholders’ accidents/illnesses that were reimbursed for imaging by the total number of policyholders’ reimbursed accidents/illnesses. Vertical lines indicate market entries of the study company’s clinics in treatment city A (5/2017) and treatment city B (8/2017)

Figure 2 displays the means of the outcomes by treatment group and over time. The levels and trends of the outcomes were reasonably similar before the market entries, suggesting that the parallel trends assumption holds. Accident patients’ probability of radiography or ultrasound (a) and any imaging (e) decreased in the market entry cities after the entries, while accident and illness patients’ MRI or CT probability (c and d) increased. The remaining plots show no post-entry differences.

Supplementary Appendix E shows that the market entries had no effect on the take-up of study company’s VPHI policies in the respective cities. Also, the age and sex composition of study company’s policyholders did not change (adverse selection). There was, however, a positive effect on the number of accidents per VPHI policyholder. Because the increase might result from VPHI policyholders getting milder injuries checked by a physician after a market entry, it is possible that my estimates of imaging overuse are biased slightly downwards.

**Fig. 3:**
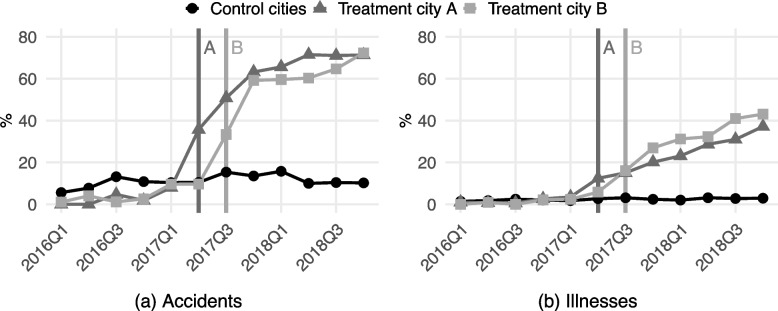
Share of policyholders’ accidents and illnesses that were treated in the study company’s clinics. *Notes:* The means were calculated by dividing the number of policyholders’ accidents/illnesses that received care in the study company’s clinics by the total number of policyholders’ reimbursed accidents/illnesses. The control group includes patients in all ten control cities. The vertical lines indicate market entries of the study company’s clinics in treatment city A (5/2017) and treatment city B (8/2017)

Lastly, Fig. 3 shows that the market entries made a substantial impact on care-seeking patterns of the study company’s policyholders. This is crucial because the estimation strategy assigns treatment to all reimbursed policyholders in the two market entry cities, even if only a portion of the patients actually received care in the study company’s clinics. The figure depicts the share of reimbursed policyholders who received care in a clinic owned by the study company (within one year of the accident or the onset of illness) and shows that the shares in market entry cities were low before the entries^16^ and increased sharply afterwards. By the end of 2018, approximately 70% (40%) of the market entry cities’ policyholders who were reimbursed for an accident (illness) received care in one of the study company’s clinics. The effect was even more marked among those who were imaged (Supplementary Appendix G Figure A2). The shares remained low and stable in the control group throughout the entire study period.

### Baseline results

**Fig. 4:**
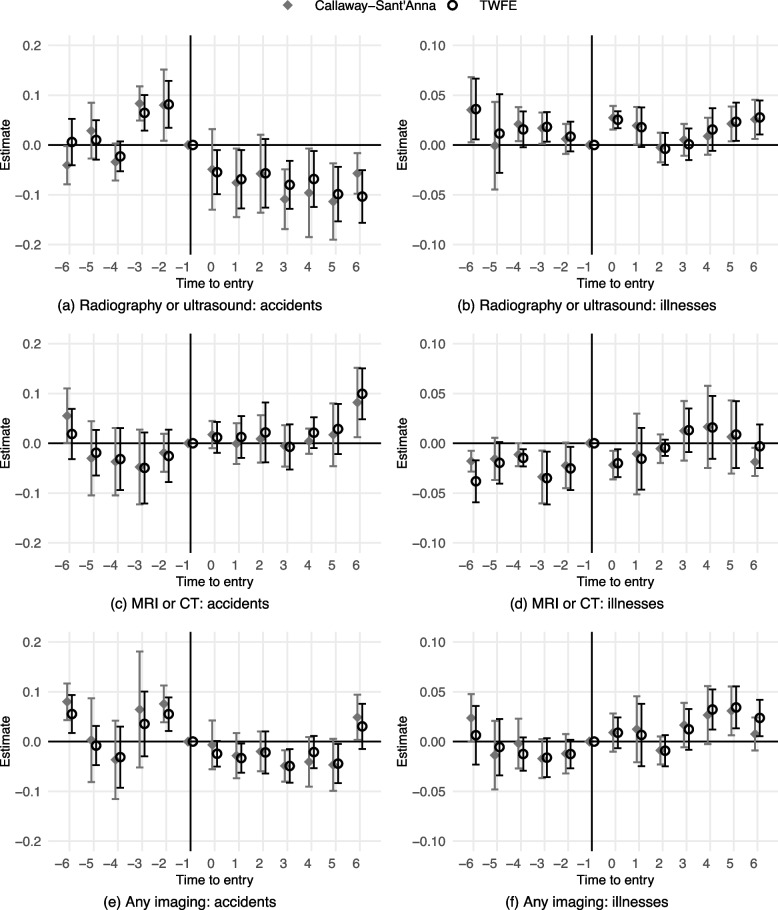
Effects of the market entries. *Notes:* 95% confidence interval. All outcomes are indicators equalling 1 whenever the sum of imaging reimbursements is ${>0}$. Estimated using Eq. (1). Standard errors are clustered at the city level (N of clusters = 12)

Figure 4 displays the baseline results. Subfigures a, c and e show results for the reimbursed accidents, while subfigures b, d, and f concern the reimbursed illnesses. Both estimators (TWFE and Callaway and Sant’Anna [65]) provide similar results. Market entries of the study company’s clinics decreased the probability of policyholders undergoing radiography or ultrasound when being treated for an accident (a), while there was no effect on the treatment of policyholders’ illnesses (b). The point estimates show a 10-percentage-point decrease among accidents, which corresponds to a 71% decrease from the mean (Table 1). Two pre-entry estimates in subfigure a are different from zero, raising concerns over a violation of the parallel trends assumption. Hence, the effect is re-evaluated with the generalised synthetic control method in the robustness tests. The probability of undergoing MRI or CT did not change because the estimates are generally not different from zero (c and d). Moreover, the overall effect on imaging is neutral, even if there are some statistically significant estimates in both samples (e and f).

To conclude, I find evidence that radiography and/or ultrasound were overused by private clinics when diagnosing insured accident (but not illness) patients. By contrast, there were no effects on MRI and/or CT. The overall effect on imaging use was also neutral.

### Effect on patients with head injury, lower back pain, knee pain and neck pain

Supplementary Appendix F Table A2 evaluates market entries’ effect on conditions that are often found to be subject to imaging overuse in the literature: traumatic head injury, non-traumatic lower back pain, non-traumatic knee pain and non-traumatic neck pain [6, 7, 9, 10, 67]. Due to small sample sizes, the results are estimated using a simpler estimation equation, where the time-to-entry indicators are replaced by one post-entry indicator. The results must be evaluated cautiously because the outcome means are noisy in small samples $\left(N=267-867\right)$; hence, the parallel trends assumption cannot be evaluated. The estimates are generally negative for radiography/ultrasound (column 2), positive for MRI/CT (column 4) and positive for any imaging (column 6). Only some of the estimates are, however, statistically significant. The conclusion from these results is that the market entries’ effects on imaging utilisation vary considerably among privately insured patients: the same imaging technology can be overused or underused depending on the medical condition.

### Monetary effects

Supplementary Appendix G Figure A3 shows the estimated effects on reimbursed expenditures (see the descriptive statistics in Supplementary Appendix F Table A3 and means of outcomes over time in Appendix G Figure A4). In line with the results concerning binary outcomes, reimbursed radiography and ultrasound expenditures decreased among VPHI policyholders who were treated for an accident (a). The magnitude is large: a decrease of €30 is as large as the mean of the variable. All the other imaging reimbursements were generally unaffected, even if accident patients’ MRI and CT reimbursements increased in the last periods (b–f). Moreover, there was no effect on overall healthcare reimbursements in either of the samples (g and h).

### Robustness tests

For the sake of simplicity, the robustness tests are estimated with a single DID estimator (rather than time-to-treatment indicators). Results for the accident sample are shown in Supplementary Appendix F Table A4 and for the illness sample in Table A5. I test whether the choice of the control group affects the results.

First, the control cities in the baseline estimations may not be comparable to the treatment cities because they are less populous, and therefore have smaller healthcare markets. I restrict the control group to the four largest control cities, and the results remain fairly similar, although the estimate for MRI/CT use among reimbursed accidents is positive and statistically significant, and estimate for any imaging among reimbursed illnesses is positive (panel A). In addition, I estimate a difference-in-differences in reverse model [68] using the more-populous capital region (in which one of the study company’s clinics opened prior to 2016) as an always-treated control group. These results are similar to those in the panel A (panel B).^17^ Second, Fig. 3 showed that a small portion of the control cities’ policyholders travelled to the study company’s clinics in other cities, and this may bias the results.^18^ Thus, I restrict the control group to the four most remote control cities (based on travel time to treatment cities by car). The estimates are, again, similar to the previous panels (panel C).

I also test whether the choices regarding econometric specification drive the baseline results. First, the baseline results do not control for the policyholders’ insurance product coverage because it might be affected by the market entries. However, controlling for the insurance product fixed effects and the level of cost-sharing does not considerably alter the results (panel D). Hence, cost-sharing does not seem to play a role in service use among insured patients unlike, for example, in Austria [69]. Second, the outcomes’ one year follow-up period spans across the market entry for accidents and illnesses that took place within one year prior to the market entry. When excluding the accidents/illnesses that took place in the four quarters preceding the market entries (from $-4$ to $-1$), the estimates are similar to the baseline results, except that the statistical significance in some estimates changes (panel E).

To further address the concerns over the optimal control group and the parallel trends assumption, I estimate the results using the generalised synthetic control method (GSCM) and constructing the control group out of all municipalities in Finland. First, I aggregate the data to a city-quarter level time series by calculating means. Second, I use the same covariates as in the baseline DID estimations—but as city-level means—to construct a synthetic control group of treatment cities A and B. Supplementary Appendix G Figure A7 displays the results.^19^ The market entries decreased radiography and ultrasound imaging in the treatment of accidents among policyholders, although only two of the estimates are statistically significant (a). Rest of the figures do not reveal effects on imaging probability (b–f).

Lastly, I test the results with alternative outcomes (Supplementary Appendix F Table A7). First, it is possible that imaging was overused by assigning the same patients to both radiography/ultrasound and MRI/CT. However, it was not found that the reform had effects on the use of multiple imaging technologies on the same patients (column 1). Second, my identification strategy relies on an assumption that the inter-city differences imaging utilisation derive from financial incentives. I test this assumption by estimating the effect on medicine reimbursements, which did not financially benefit any clinic or physician because legislation prevents them from being financially involved in the medicine business.^20^ I find no effects (column 2).

## Discussion

I have studied the overuse of healthcare services among VPHI policyholders who were treated in Finland’s privately owned clinics. I used administrative claims data from a major Finnish insurance company and based the identification strategy on the market entries of clinics owned by the insurance company. The previous literature has found that the overuse of medical imaging is common across technologies and medical conditions, and magnitudes above 50% are not uncommon [7–10]. Moreover, the overuse is potentially more common in Finland’s private clinics than in other settings, because the clinics typically treat the healthier part of the population [70], who can be less likely to need the services. This study uncovered supporting evidence of radiography and ultrasound overuse among privately insured patients who were treated for accidents, although not among treatment for illnesses. Moreover, there was no evidence on MRI and CT overuse among treatment of accidents or illnesses. An inspection of commonly studied medical conditions (such as non-traumatic lower back pain) revealed considerable variation across medical conditions, although strong conclusions cannot be made due to the small sample sizes. Lastly, I showed that the monetary effects were similar to the baseline results: payer-provider integration resulted in cost-containment only through reduced radiography and ultrasound reimbursements; however, nevertheless, they did not translate to a reduction of total reimbursements.

There is also a large body of literature estimating the effects of physician/provider financial incentives on the use of healthcare services. Higher reimbursements in a fee-for-service scheme generally increase service use [26], including imaging services [13]. My findings are mixed and show that financially motivated healthcare overuse varies depending on the imaging technology. The fact that the overuse exists in less-advanced imaging technologies (radiography and ultrasound) rather than in more advanced ones (MRI and CT) is not unintuitive: radiation doses are lower (although they are nonexistent in MRI), the examinations are cheaper and faster, and the devices are abundant. Hence, the overuse of less-advanced imaging technologies is easier and less controversial than the overuse of more-advanced ones.

The literature on the effects of payer-provider integration on cost-containment provides mixed results [33–37]. This study shows that the integration did not result in cost-containment under free choice of clinic. On the other hand, it shows that integration affected physicians’ treatment decisions by decreasing the probability of radiography and ultrasound imaging. This change, however, did not decrease total imaging expenditures. Thus, more research is needed in order to determine the necessary conditions for payer-provider integration to effectively curb the rise of healthcare expenses.

There were five main limitations in the study. First, because clinics are never established randomly, the opening of the insurance company’s clinics to treatment cities A and B was necessarily endogenous. This setting corresponds to quasi-experimental studies that estimate effects of regional reforms, because the reform regions are almost always chosen non-arbitrarily by policymakers. In this case, the study company’s clinics were established in areas that had (i) the best supply of private and public healthcare as well as (ii) the largest population (i.e. demand for healthcare). Based on the geographical distribution of the population (Supplementary Appendix A), it is reasonable to expect that the study company also aimed at maximising the availability of its services across the country. Although the non-random selection into the treatment group is likely to affect the results, several factors suggest that there is only a small bias in the results. The baseline control group consists of cities that potentially had lower supply and demand than the treatment cities. However, the results are similar when using the capital region—which had the largest supply for healthcare in the country according to the statistics in Supplementary Appendix A—as a control group. Also, it was extremely unlikely that the cities were chosen based specifically on demand and supply of *imaging* services, which make up only a small part of the reimbursed expenditures (see Supplementary Appendix F Table A3). Neither was it likely that more morbid patients selected themselves into the treatment group by choosing to migrate to market entry cities, because all cities had private healthcare services available.

Second, the identification of overuse relies on the assumption that providers overused imaging for VPHI policyholders only if they had financial incentives to do so. Although it is highly unlikely that the clinics differed in, for example, physicians’ knowledge or patients’ expectations, publicly available information does not allow to completely rule them out. Third, the data do not allow for separating CT scans from MRI scans or radiographs from ultrasound scans, which may dilute the estimated effects if the market entries had opposing effects on the use of different imaging technologies. For example, MRI and CT markets may have differences in responses to the market entries because duration of MRI is considerably longer and, hence, it may be subject to longer waiting times than CT. Fourth, it was not possible to assess the effects on VPHI policyholders’ health outcomes due to a lack of data.

## Conclusions

The research contributed to the literature by employing a new identification strategy and found that private clinics overused radiography (similar to Chalkley and Listl [31] and Kalmus et al. [32] in the dental setting) and/or ultrasound when treating patients with a voluntary private health insurance. There was, however, no overutilisation of MRI and CT (unlike in Zabrodina et al. [29] and Ikegami et al. [30]). The policy implication is that the existence and magnitude of the imaging overuse depends greatly on the insured patient’s medical ailment and the imaging technology that is typically employed to that ailment, as well as underlying characteristics of the imaging markets, such as the capacity constraints. This variance highlights the need for further research on the non-financial determinants of overuse. My results suggest that even if the discussion of imaging overuse often concentrates on the more-advanced imaging technologies, overuse of less-advanced technologies may also exist, but it can fly under the radar because cheaper and faster services are less controversial to overuse. I conclude that the payer-provider integration has a potential to affect imaging overuse.

## Supplementary Information

Supplementary Material 1.

## Data Availability

The study company does not provide access to the claims data to other researchers. The statistical code is available from the author upon request.

## Abbreviations

CT: Computed tomography
DID: Difference-in-differences
GP: General practitioner
GSCM: Generalised synthetic control method
MRI: Magnetic resonance imaging
NHS: National Health Service
TWFE: Two-way fixed effects
VPHI: Voluntary private health insurance

## Footnotes

1 In France, for example, health insurance has increased healthcare overprovision [15].

2 Payer-provider integration can also be pursued through contracts and collaboration between payers and providers, as is typically the case in the United States [16, 17].

3 A possible source of selection is, for example, patients’ tendency to choose a clinic based on the severity of their condition. Another likely source of selection is that clinics without imaging equipment refer patients to the study company’s clinics, depending on their severity.

4 In the literature, low-value imaging is often used interchangeably with inappropriate imaging use and imaging overuse.

5 Unlike many papers reviewed by Léonard et al. [24], the study by Gottschalk et al. [28] did not find a correlation between physician density and overprovision.

6 In the United States, only a small share of health management organisations (HMOs) have been staff-model HMOs, which employ their own physicians rather than contract with physicians and hospitals [16, 17].

7 In order to steer the treatment choices of physicians working in the study company’s clinics, the company implemented multiple measures, such as guidance and criteria for diagnostics [36].

8 In 2016–2018, patient fees of physician appointments in public primary care were capped at approximately €60 per person per *year* [39].

9 For example, the price of a CT scan on the head in one of the three largest private clinic chains was €402 out of which NHI reimbursed €39. Hence, the copayment was €363 [40].

10 Finland has five university hospitals, which are specialised (i.e. secondary) care providers that have the best resources and the widest treatment portfolio among all hospitals in the country. The study company’s clinics were established in the same five cities where the university hospitals were located. In addition, these five cities had the largest number of incumbent private clinics before the market entries. In addition to supply factors, the study company’s clinics were established in areas with the greatest demand, as the areas were also among the seven largest ones by population (if the capital region is considered as one area). By comparison, more than half of the Finnish municipalities did not have any private clinics, likely because their sparse population is not able to sustain private healthcare provision on top of the public healthcare. See supplementary Appendix A for more information.

11 There is some evidence that the goals were met, as the occupational healthcare patients had shorter sick leave periods and a lower probability of surgery in the study company’s clinics than in other clinics, although the findings may result from selection into clinics [36].

12 Appointment, radiography, laboratory tests, etc.

13 The data include the (patient-reported) date of accident/illness and the date when reimbursement was paid, but not the date of service provision. Hence, the follow-up period is based on the timing of the reimbursement rather than the timing of the service. In supplementary Appendix C, I show that one year is a sufficient follow-up period to cover most of the imaging reimbursements. In addition, policyholders did not have incentives to report an incorrect date.

14 I also test whether the patient characteristics change due to the market entries (supplementary Appendix E).

15 The study setting complies with the Callaway-Sant’Anna identification assumptions because (i) the treatment is irreversible and (ii) trends are parallel based on the “never-treated” and “not-yet-treated” groups (see the section on descriptive statistics). The study’s Callaway-Sant’Anna estimates use the “never-treated” units as the counterfactual.

16 They were not zero because the study company had opened clinics in other cities prior to 2017, and patients were allowed to choose them if they preferred to travel to those clinics.

17 In the supplementary Appendix F Table A6 displays the descriptive statistics of the outcomes. Appendix G Figure A5 shows that the required *post*-treatment parallel trends assumption [68] generally holds. In addition, Appendix G Figure A6 shows that a considerable share of the capital region’s accident patients were treated in the study company’s clinics during the whole study period.

18 If, for example, the most morbid patients from the control cities were most likely to start travelling to the study company’s clinics after the market entries, the estimates would be biased.

19 After aggregating the data to the city-quarter level, I exclude observations that were based on fewer than 20 patients. The sample size is 387 in the accident sample and 1, 124 in the illness sample.

20 Only pharmacies can sell medicines, and only university-educated pharmacists (not firms or non-pharmacists) can own pharmacies [18].
