# Supplementary material for: Overuse of medical imaging and effects of payer-provider integration: quasi-experimental evidence from Finland
Source: Health Econ Rev. 2025 Jan 28;15:4. doi: 10.1186/s13561-025-00592-0 (PMC11776152; doi:10.1186/s13561-025-00592-0)
Supplement: Supplementary file 1 — Supplementary Material 1. [file 13561_2025_592_MOESM1_ESM.pdf]

ONLINE APPENDIX

Health Economics Review

Overuse of medical imaging and payer-provider integration: quasi-experimental evidence from Finland

Konsta Lavaste[1,2]

[1]Finnish Institute for Health and Welfare, PO Box 30, 00271, Helsinki, Finland
[2]University of Jyväskylä, PO Box 35, 40014, Jyväskylä, Finland

Appendix A. Construction of the control group

Finland is a sparsely populated country: its land area is almost the size of Germany, whereas Germany's population is 15 times larger than Finland's. The figure below displays a map of Finland divided into municipalities. The most populated municipalities are called cities. Each municipality is represented by a bubble that matches its population size. The colour of the bubble shows which municipalities we include in this study. The figure highlights that the Finnish population is concentrated in a small number of municipalities—i.e. the largest cities—while the rest of the municipalities are sparsely populated.

Because of the dispersion of the population, the markets for private healthcare services differ significantly among municipalities. The next figure further highlights the differences in municipal healthcare markets by showing how many private clinics each municipality had in 2017. More than half of the municipalities did not have any private clinics, while 16 municipalities had at least 4 clinics. The clinics are concentrated on the larger municipalities, that is, the cities. Despite having municipality-level data on imaging capacity, I expect that the municipalities' imaging capacity correlates heavily with the number of imaging units. Thus, the private imaging markets are likely to be similar across similar-sized cities.

This analysis highlights that it is likely that the majority of the municipalities are not comparable to the two cities into which the study company opened its clinics. More precisely, the inhabitants in most of the municipalities have very limited possibilities to use private healthcare services. Therefore, it is not desirable to use all patients outside the market entry cities as the control group.

In the baseline analysis, I have chosen to construct the control group from the patients from the 10 largest cities (which did not have the study company's clinic). Even if the number of cities is arbitrary, this approach is justifiable because these cities had (i) at least three private clinics each and (ii) a similar socioeconomic structure. The socioeconomic structure plays a major role in the private healthcare market, because private services are used especially by higher socioeconomic classes.[21] The table below displays the characteristics of the largest cities. The characteristics are fairly similar and generally more favourable to private healthcare use than in Finland on average.

[21]Blomgren, J. & Virta, L.J. (2020). Socioeconomic differences in use of public, occupational and private health care: A register-linkage study of a working-age population in Finland. PLoS ONE 2020;15(4):1–18. https://doi.org/10.1371/journal.

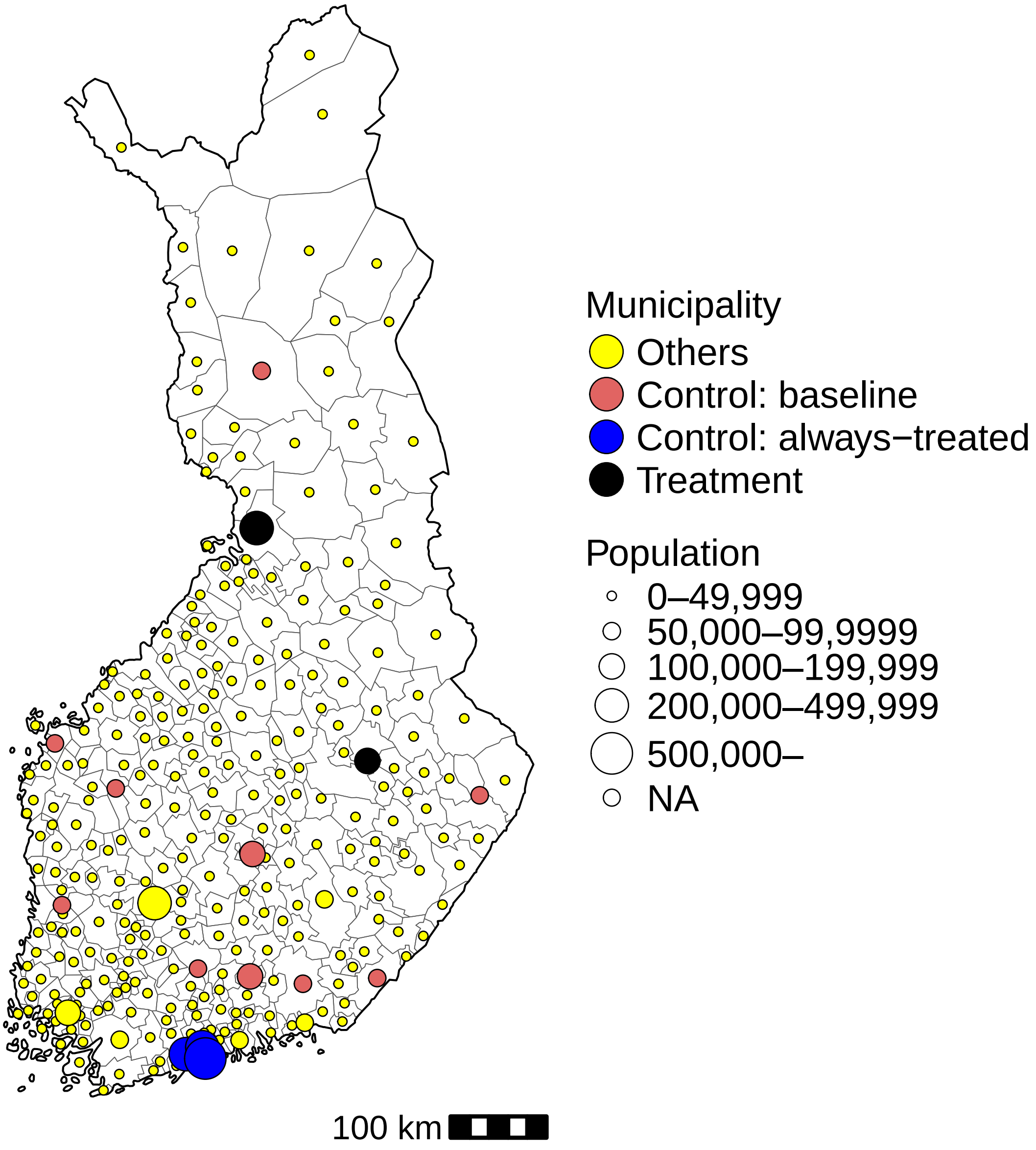

Fig. Number of inhabitants. *Notes:* Figure depicts the map of Finland (excluding Åland islands). Bubble size depicts the number of inhabitants. Black lines indicate municipality borders in 2017. Source: Statistics Finland (`https://pxdata.stat.fi/PxWeb/pxweb/en/Kuntien_avainluvut/` [accessed on 25 September 2024]).

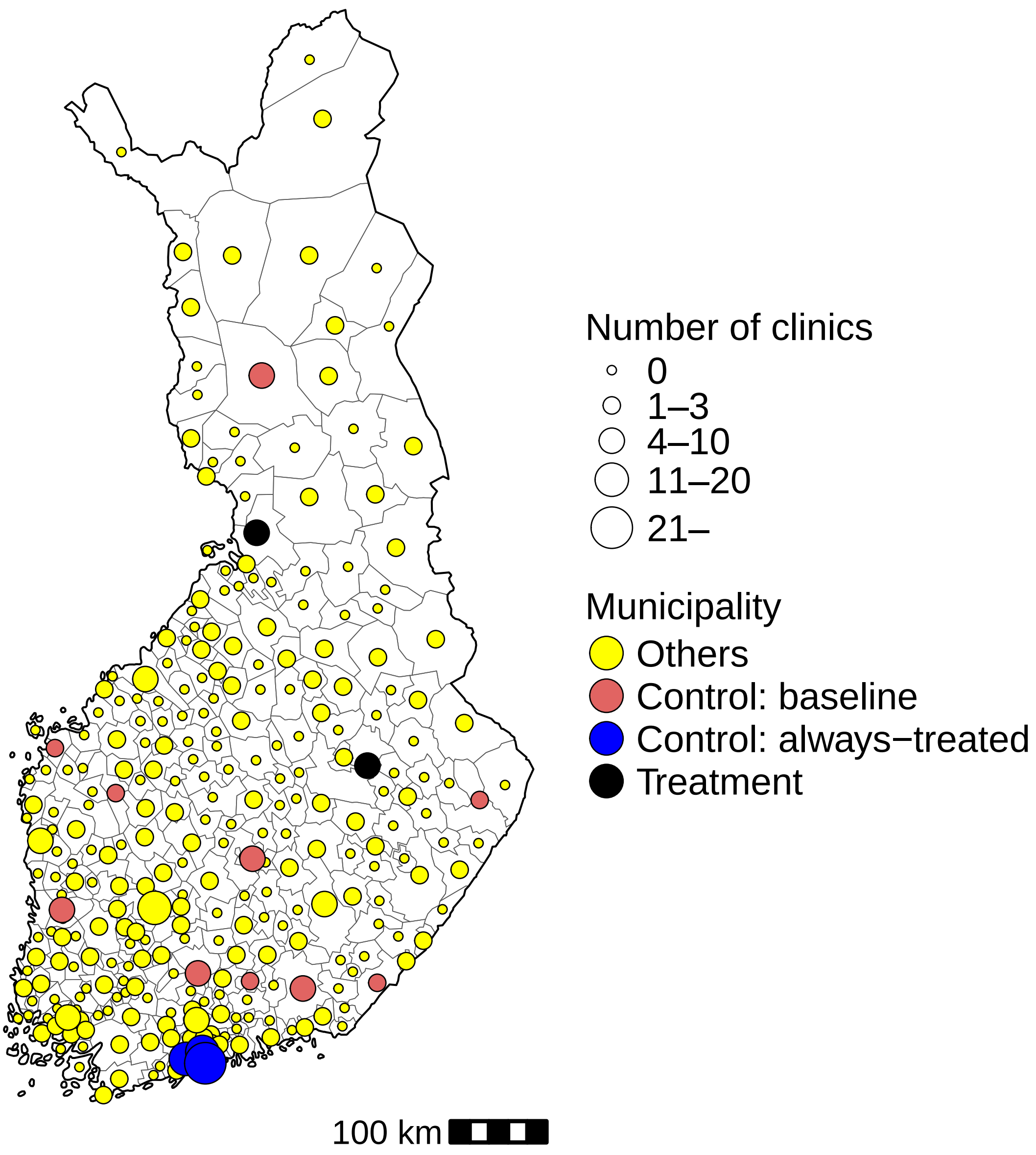

Fig. Number of private clinics by municipality in 2017. *Notes:* Figure depicts the map of Finland (excluding Åland islands). Black lines indicate municipality borders in 2017. Source: Lavaste K. Private primary care clinics in Finland in 2016–2018. 2023. `https://doi.org/10.17632/dwgrkmf7rg.1`.

Table Characteristics of the largest cities

Rank by population	Group[a]	Market entry	Population (1,000 persons)[b]	Working-age (%)[c]	Foreign nationals (%)[d]	Population density (ppl/km^2)[e]	Morbidity index[f]	Median income (€10K)[g]	N of private clinics[h]
1	R	2013	643	68.9	9.5	3002	84	37	33
2	R		279	66.2	10.5	894	77	47	12
3		2016	232	67.5	4.6	442	96	31	17
4	R		223	67.0	11.2	936	87	40	14
5	T_A	2017	202	65.9	2.9	68	121	35	8
6		2018	190	66.7	6.5	772	102	30	8
7	C		140	66.4	3.3	120	110	32	4
8	C		120	61.8	4.3	260	111	31	3
9	T_B	2017	118	64.6	2.5	37	133	33	9
10	C		85	60.6	2.3	73	111	33	4
11	C		84	59.6	2.7	33	107	34	4
12	C		76	65.0	3.1	32	122	30	3
13	C		73	62.9	4.5	51	99	32	3
14	C		68	60.3	3.7	38	100	35	4
15	C		67	64.6	6.0	185	94	34	3
16	C		63	63.3	1.7	44	104	35	3
17	C		62	64.9	2.5	8	109	33	4
Whole country			5,513	59.3	4.5	18	100	36	309

Notes: Includes all Finnish municipalities (i.e. cities) which had more than 60,000 inhabitants in 2017.

[a] T_A = treatment city A, T_B = treatment city B, C = control city, R = always-treated control city (in the capital region) in the reverse DID estimations.

[b] Population in 2017. Source: Official Statistics of Finland (OSF): Population structure [e-publication]. ISSN=1797-5395. Helsinki: Statistics Finland [referred: 3.10.2022]. Access method: `http://www.stat.fi/til/vaerak/meta_en.html`.

[c] 18–64-year-olds. Source: Official Statistics of Finland (OSF): Population structure [e-publication]. ISSN=1797-5395. Helsinki: Statistics Finland [referred: 3.10.2022]. Access method: `http://www.stat.fi/til/vaerak/meta_en.html`.

[d] Source: Official Statistics of Finland (OSF): Population structure [e-publication]. ISSN=1797-5395. Helsinki: Statistics Finland [referred: 3.10.2022]. Access method: `http://www.stat.fi/til/vaerak/meta_en.html`.

[e] Source: Official Statistics of Finland (OSF): Population structure [e-publication]. ISSN=1797-5395. Helsinki: Statistics Finland [referred: 3.10.2022]. Access method: `http://www.stat.fi/til/vaerak/meta_en.html`.

[f] Age-standardised morbidity index. 100 = whole country. Source: Finnish Institute for Health and Welfare. Accessed from Sotkanet database: `https://sotkanet.fi/sotkanet/en/metadata/indicators/243`.

[g] Median disposable household income in tens of thousands of euros. Source: Official Statistics of Finland (OSF): Taxable incomes [e-publication]. Helsinki: Statistics Finland [referred: 3.10.2022]. Access method: `http://www.stat.fi/til/tvt/meta_en.html`.

[h] Absolute number of private clinics. Source: Lavaste K. Private primary care clinics in Finland in 2016–2018. 2023. `https://doi.org/10.17632/dwgrkmf7rg.1`).

Although I argue that the control group of 10 largest cities is the most suitable for this analysis, I implement robustness estimations with alternative control groups. I (i) keep only the four most populated cities in the control group, (ii) keep only the four most remote cities (out of the 10 baseline cities) in the control group, and (iii) use the capital region's cities as an always-treated control group. Lastly, I (iv) aggregate the data to the city level and construct a synthetic control group out of all Finnish municipalities (excluding market entry cities). These results are estimated with the generalized synthetic control method.

Appendix B. Market entries

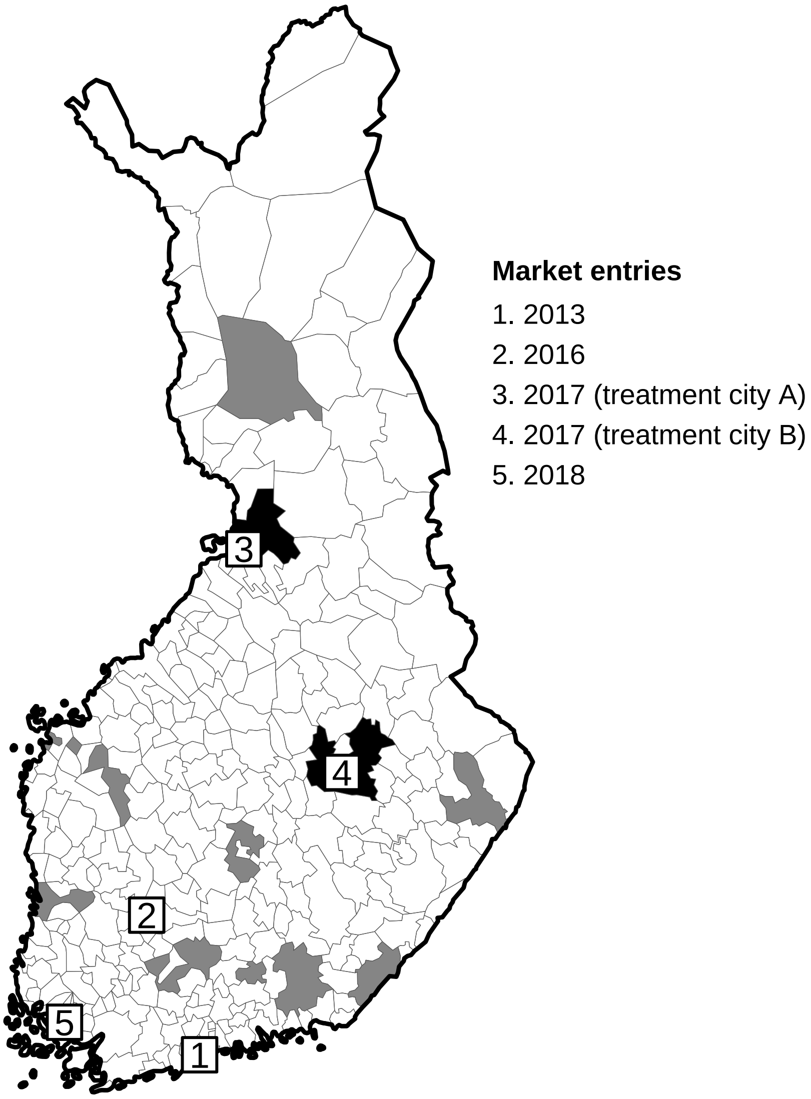

Fig. Market entries of the study company's clinics. *Notes:* Borders indicate municipalities in 2017. The study company's clinics marked with numbers 1–5. Black areas are the two municipalities (cities) in which the study company opened its clinics in 2017. Grey areas are the baseline control municipalities (cities). In total, there were 295 municipalities in 2017.

Appendix C. Length of the follow-up period

The claims data include the month of accident/illness and the month of reimbursement—but not the month when the imaging took place. Because the data are available only for 2016–2019, I restrict the sample to claims paid within one year from the time of the accident or the onset of illness in order to make accident-/illness-level observations comparable. The downside of this is that claims with, for example, long processing times were unintentionally left out of the samples.

Cumulative distributions of imaging claims by the time from the accident/illness to the payment of the claim are shown below. The figure includes only claims of accidents/illnesses that took place in 2016, allowing for a 3–4-year follow-up period. Subfigure a shows that approximately 85% of radiography and ultrasound claims were paid within one year of the time of an accident or the onset of an illness. In other words, I exclude 15% of the radiography and ultrasound claims by restricting the sample to the one-year follow-up period. A portion of these excluded claims are excluded intentionally because the imaging was actually performed more than one year after the accident/illness, and another portion are excluded unintentionally because the imaging was performed within one year but, for some reason, the claim was not paid within one year. Nevertheless, 15% is not a considerable share in this setting, especially because some of the excluded claims concern subsequent imaging of the same patient. The baseline outcomes are binary indicators and, thus, not affected by the subsequent imaging.

The conclusion is that a one-year follow-up period is sufficiently long to include most of the imaging claims. Processing times might affect identification if there were systematic differences between cities; however, this is unlikely because claims are submitted online or directly by providers, most of which were clinic chains operating in all treatment and control cities. It is possible, however, that claims related to care in the study company's clinics were processed faster than claims related to other private clinics. This means that patients who visited the study company's clinics received, on average, more reimbursements within one year than patients who visited other private clinics. Hence, the post-entry probability of imaging is artificially higher in the market entry cities than in the control cities—causing an upward bias in the estimates and underidentification of overuse (or overidentification of underuse).

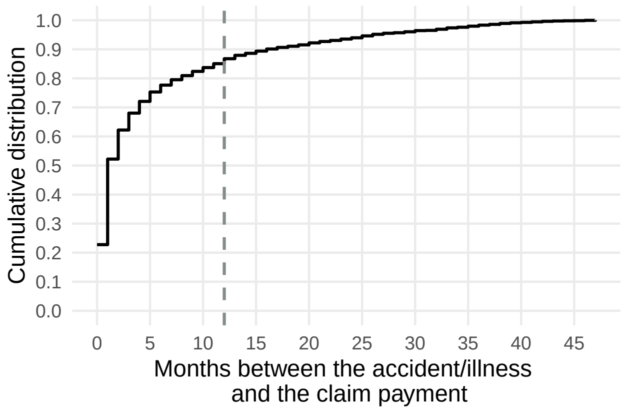

(a) Radiography or ultrasound

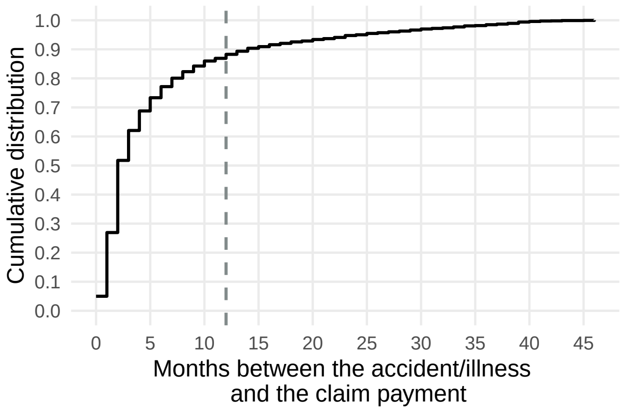

(b) MRI or CT

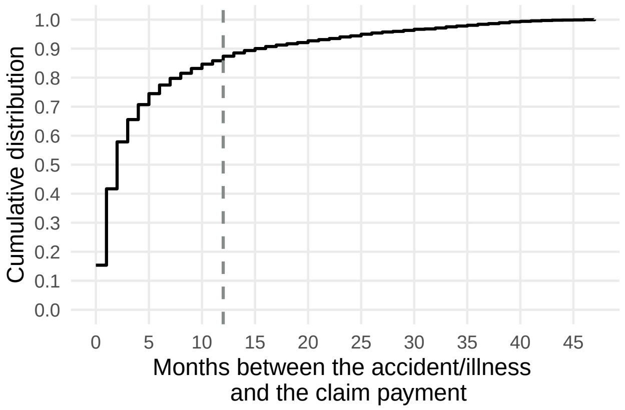

(c) All imaging

Fig. Cumulative distribution of claims by months between accident and payment. *Notes:* Plots include all radiography/ultrasound claims (a), MRI/CT claims (b) and radiography/ultrasound/MRI/CT claims (c) that took place in 2016. Dashed vertical line indicates one year.

Appendix D. Data

Nationwide register data from a Finnish insurance company were employed. The data include all claims on self-purchased non-statutory health insurance policies paid by the study company in 2016–2019. The sample was divided into two main samples and four additional samples using variables on (i) the cause of the healthcare expense and (ii) the associated medical condition:

Table Samples

	Sample	Description
Panel A. Main samples		
(1)	Accidents	All claims in which the cause of healthcare costs was an accident
(2)	Illnesses	All claims in which the cause of healthcare costs was an illness
Panel B. Additional samples		
(3)	Accidental head injuries	All claims in which the cause of healthcare costs was an accident and the associated medical condition was head injury
(4)	Non-accidental lower back pain	All claims for which the cause of healthcare costs was an illness and the associated medical condition was lower back pain
(5)	Non-accidental knee pain	All claims for which the cause of healthcare costs was an illness and the associated medical condition was knee pain
(6)	Non-accidental neck pain	All claims for which the cause of healthcare costs was an illness and the associated medical condition was neck pain

After identifying the samples, I excluded claims with negative reimbursement value (i.e. deductibles) and claims paid after one year from the accident or the onset of illness. Next, claims were grouped by their accident/illness identifier and four main outcomes and four additional outcomes were constructed:

Table Outcome variables

	Outcome	Description
Panel A. Main outcomes		
(1)	Radiography or ultrasound (0/1)	Indicator equalling 1 if the monetary sum of radiography and ultrasound reimbursements over the accident/illness identifier was greater than zero.
(2)	MRI or CT (0/1)	Indicator equalling 1 if the monetary sum of MRI and CT reimbursements over the accident/illness identifier was greater than zero.
(3)	Any imaging (0/1)	Indicator equalling 1 if the monetary sum of all imaging reimbursements over the accident/illness identifier was greater than zero.
Panel B. Monetary outcomes		
(4)	Radiography or ultrasound (€)	Monetary sum of radiography and ultrasound reimbursements over the accident/illness identifier.
(5)	MRI or CT (€)	Monetary sum of MRI and CT reimbursements over the accident/illness identifier.
(6)	All imaging (€)	Monetary sum of all imaging reimbursements over the accident/illness identifier.
(7)	All expenditures (€)	Monetary sum of all reimbursements over the accident/illness identifier
Panel C. Alternative outcomes		
(8)	Both radiography or ultrasound and MRI or CT (0/1)	Indicator equalling 1 if the monetary sum of radiography and ultrasound reimbursements over the accident/illness identifier was greater than zero and the monetary sum of MRI and CT reimbursements over the accident/illness identifier was greater than zero.
(9)	Medicines (€)	Monetary sum of medicine reimbursements over the accident/illness identifier.

Next, the data were aggregated to the level of accident/illness identifier. The resulting data were restricted to accidents which took place in 2016–2018 or illnesses which began in 2016–2018. The resulting data include all accidents and illnesses with an equally long (one-year) follow-up period.

The empirical strategy is based on comparing patients in a specific set of cities (i.e. largest municipalities). Hence, the samples were further restricted to patients who resided either in the two market entry cities (A or B) or in the 10 control cities at the time of the accident or the onset of an illness. Because there were some municipality mergers during the time period (some of which affected our treatment and control cities), the municipality classification was fixed to 2018. Also excluded were patients for whom age or gender was missing or who were younger than 18 years old or older than 64 years old at the time of the accident or the onset of the illness. Further excluded were (i) accidents/illnesses which were reimbursed for any healthcare expenditures outside Finland or (ii) which were reimbursed *only* for medicine or physiotherapy expenditures (if the patient did not see a physician, the probability of imaging was zero).

The estimation equation (1) includes the following variables:

Table Variables related to the estimation

	Variable	Description
(1)	Treated_m (0/1)	Treatment group indicator equalling 1 if m is treatment city A or B.
(2)	Post_{tm} (0/1)	Post-entry indicator equalling 1 if either m is treatment city A and $t \geq$ Q2/2017 or m is treatment city B and $t \geq$ Q3/2017.
(3)	$\sum_{a=-6}^{+6} \mathbb{1}[t - t_m^{\text{entry}} = a]$ (0/1)	12 time-to-entry indicators equalling 1 if the time of the accident or the onset of the illness (t) minus city-specific market entry timing (t_m^{entry}) equals the time-to-entry index (a).

Lastly, a set of binary indicators to control patient characteristics $\mathbf{X}_{it}$ were constructed:

Table Control variables

	Variable	Description
Panel A. Baseline controls		
(1)	Female (0/1)	Indicator equalling 1 if the patient was female.
(2)	Age bins (0/1)	Indicators equalling 1 if the patient was either 18–27, 28–37, 38–47, 48–57, or 58–64 years old at the time of the accident or the onset of the illness.
Panel B. Additional controls		
(3)	Level of cost-sharing (€)	Monetary sum of paid deductibles over the accident/illness identifier

Appendix E. Effects of market entries on the number, composition and behaviour of insured patients

In this section, I rule out the possibility that the market entries of the study company's clinics affected the number, composition or behaviour of insured patients in a way which would drive the main results. I estimate a simple difference-in-differences specification using city-year-level data in 2016–2018 (3 time points and 12 cities = 36 observations):

$$y_{mt} = \delta_0 + \delta_1 \text{Post}_t \times \text{Treated}_m + \lambda_t + \mu_m + \varepsilon_{mt}, \tag{A1}$$

where Treated_m is the treatment variable, which equals 1 for treatment cities A and B. Post_t equals 1 if $t = \{2017, 2018\}$. μ_m are the city fixed effects (which absorb Treated_m); they take into account time-invariant population characteristics, such as age structure, income and education level. λ_t are the time fixed effects which absorb Post_t. I cluster the standard errors at the city level (N = 12). The DID estimate δ_2 indicates whether the entry exerted an effect on the outcome in the treatment cities.

The table below displays the estimated effects. Market entries did not increase voluntary private health insurance take-up in the treated cities relative to the control cities because the estimates are statistically insignificant and small in magnitude (column 1). The mean age of patients (in the claims data) did not increase (column 2), although the share of females grew slightly (column 3). The magnitude is, however, very small: less than 1% in comparison to the pre-entry mean. Lastly, the market entries had a positive effect on the number of reimbursed accidents (but not illnesses) per insured person in the market entry cities. This is natural because the market entries increased the availability of healthcare services in the respective cities.

Table Effect of market entries on insurance take-up, insuree characteristics and number of accidents/illnesses

		Insuree characteristics		
	Insurance take-up[a]	Mean age of insured persons	Share of female insured persons[b]	N of accidents/illnesses per insured person[c]
	(1)	(2)	(3)	(4)
Panel A. Accidents				
$\text{Post}_t \times \text{Treated}_m$	-0.006	0.086	0.002	0.004**
	(0.003)	(0.139)	(0.001)	(0.001)
N	36	36	36	36
$\text{mean}(y_{mt} \| Post_t = 0)$	0.164	37.499	0.528	0.022
Panel B. Illnesses				
$\text{Post}_t \times \text{Treated}_m$	0.002	0.140	0.005*	0.008
	(0.002)	(0.152)	(0.002)	(0.008)
N	36	36	36	36
$\text{mean}(y_{mt} \| Post_t = 0)$	0.070	37.602	0.525	0.180
City FEs	✓	✓	✓	✓

Notes: t-test level of significance: *< 0.05, **< 0.01, ***< 0.001. Standard errors in parentheses. Estimated using equation (A1). Includes city-year level observations in 2016 (pre-entry) and 2017–2018 (post-entry). The outcomes concern the situation in 31st December each year, except for N of accidents/illnesses which is the count for the whole year. Standard errors clustered at the city level (N of clusters = 12).
[a] N of insured persons aged 18–64 divided by 18–64-year-old population in city m in year t. City populations from Statistics of Finland: Paavo postal code area statistics (`https://www.stat.fi/tup/paavo/index_en.html` [accessed on 21 May 2023]).
[b] N insured females divided by N of all insured persons in city m in year t.
[c] N of accidents/illnesses divided by number of persons with accident/illness coverage in city m in year t.

Appendix F. Additional tables

Table A1 Summary statistics: Patient characteristics and relative group sizes

	Mean	SD	Min	Max	N
Panel A. Accidents					
Age: 18–27	0.25	0.44	0	1	8,496
Age: 28–37	0.26	0.44	0	1	8,496
Age: 38–47	0.10	0.30	0	1	8,496
Age: 48–57	0.17	0.37	0	1	8,496
Age: 58–64	0.15	0.35	0	1	8,496
Female	0.50	0.50	0	1	8,496
Treatment city A	0.24	0.43	0	1	8,496
Treatment city B	0.13	0.34	0	1	8,496
Control cities	0.63	0.48	0	1	8,496
Panel B. Illnesses					
Age: 18–27	0.25	0.43	0	1	29,332
Age: 28–37	0.30	0.46	0	1	29,332
Age: 38–47	0.10	0.30	0	1	29,332
Age: 48–57	0.14	0.35	0	1	29,332
Age: 58–64	0.13	0.34	0	1	29,332
Female	0.72	0.45	0	1	29,332
Treatment city A	0.15	0.36	0	1	29,332
Treatment city B	0.11	0.32	0	1	29,332
Control cities	0.73	0.44	0	1	29,332

Notes: Includes accident-/illness-level observations in 2016–2018.

Table A2 Effect of market entries on imaging likelihood in additional samples

	Radiography or ultrasound	MRI or CT	Any imaging
	(1)	(2)	(3)
Panel A. Traumatic head injury			
$\text{Post}_{tm} \times \text{Treated}_m$	0.038	0.158*	0.178*
	(0.031)	(0.067)	(0.069)
N	267	267	267
mean(y_{imt})	0.022	0.139	0.157
Panel B. Non-traumatic lower back pain			
$\text{Post}_{tm} \times \text{Treated}_m$	-0.048	0.136**	0.112*
	(0.034)	(0.034)	(0.050)
N	867	867	867
mean(y_{imt})	0.065	0.391	0.437
Panel C. Non-traumatic knee pain			
$\text{Post}_{tm} \times \text{Treated}_m$	-0.015	0.025	0.078
	(0.036)	(0.079)	(0.088)
N	449	449	449
mean(y_{imt})	0.094	0.546	0.608
Panel D. Non-traumatic neck pain			
$\text{Post}_{tm} \times \text{Treated}_m$	-0.100	-0.126*	-0.149
	(0.049)	(0.055)	(0.105)
N	442	442	442
mean(y_{imt})	0.084	0.195	0.258
City & time FEs	✓	✓	✓
Age & sex	✓	✓	✓
Accident/illness FEs	✓	✓	✓

Notes: t-test level of significance: *$<$ 0.05, **$<$ 0.01, ***$<$ 0.001. Standard errors in parentheses. Outcomes: indicator equalling 1 whenever the sum of imaging reimbursements > 0. Includes accident-/illness-level observations for working-age adults (18–64 years old) in 2016–2018. Standard errors clustered at the city level (N of clusters = 12).

Table A3 Summary statistics: Monetary outcomes

	Mean	SD	Min	Max	N
Panel A. Accidents					
Radiography or ultrasound (€)	30.28	93.92	0	1,534	8,496
MRI or CT (€)	83.13	163.27	0	1,518	8,496
Any imaging (€)	113.42	186.47	0	1,707	8,496
All expenses (€)	885.78	2875.02	1	138,605	8,496
Panel B. Illnesses					
Radiography or ultrasound (€)	23.02	79.98	0	1,358	29,332
MRI or CT (€)	24.10	107.56	0	5,499	29,332
Any imaging (€)	47.12	134.55	0	5,499	29,332
All expenses (€)	418.83	1032.34	2	47,736	29,332

Notes: Includes accident-/illness-level observations in 2016–2018. Outcomes are based on claims within one year from the accident or the onset of illness.

Table A4 Robustness tests: Accidents

	Radiography or ultrasound (1)	MRI or CT (2)	Any imaging (3)
Panel A. Only the four largest control cities[a]			
$\text{Post}_{tm} \times \text{Treated}_m$	-0.087***	0.072**	-0.007
	(0.011)	(0.017)	(0.011)
N	6,030	6,030	6,030
mean(y_{imt})	0.135	0.253	0.364
Panel B. Difference-in-differences in reverse[b]			
$\text{Post}_{tm} \times \text{Treated}_m$	-0.076**	0.036**	-0.019
	(0.012)	(0.005)	(0.008)
N	14,177	14,177	14,177
mean(y_{imt})	0.143	0.263	0.382
Panel C. Only the four most remote control cities[c]			
$\text{Post}_{tm} \times \text{Treated}_m$	-0.110***	-0.007	-0.084**
	(0.015)	(0.016)	(0.017)
N	4,615	4,615	4,615
mean(y_{imt})	0.124	0.247	0.35
Panel D. Controls for insurance product characteristics[d]			
$\text{Post}_{tm} \times \text{Treated}_m$	-0.100***	0.037	-0.048*
	(0.014)	(0.019)	(0.021)
N	8,496	8,496	8,496
mean(y_{imt})	0.136	0.244	0.356
Panel E. Excluding accidents which took place within one year prior to the entries[e]			
$\text{Post}_{tm} \times \text{Treated}_m$	-0.084***	0.029	-0.037
	(0.005)	(0.019)	(0.022)
N	7,581	7,581	7,581
mean(y_{imt})	0.13	0.246	0.353
City and time FEs	✓	✓	✓
Age and sex	✓	✓	✓
Accident/illness FEs	✓	✓	✓

Notes: t-test level of significance: *$<$ 0.05, **$<$ 0.01, ***$<$ 0.001. Standard errors in parentheses. Outcomes: indicator equalling 1 whenever the sum of imaging reimbursements > 0. Standard errors are clustered at the city level. N of clusters in panels B, D and E = 12. N of clusters in panels A and C = 6.

[a] Includes only four (out of ten) largest control cities by population.

[b] The capital region as the always-treated control group.

[c] Includes only four (out of ten) control cities which are the most remote. I retrieved Google Maps driving times from each control city to both treatment cities by car, and used the smaller of the two values as the measure of remoteness (i.e. the driving time to the nearest treatment city).

[d] Added (i) control for the level of cost-sharing and (ii) insurance product fixed effects.

[e] Excluding observations for which $\text{Treated}_m = 1$ and the time (quarters) to treatment equals -1, -2, -3, or -4.

Table A5 Robustness tests: Illnesses

	Radiography or ultrasound (1)	MRI or CT (2)	Any imaging (3)
Panel A. Only the four largest control cities[a]			
$\text{Post}_{tm} \times \text{Treated}_m$	0.004	0.015	0.022*
	(0.003)	(0.007)	(0.007)
N	20,149	20,149	20,149
mean(y_{imt})	0.098	0.068	0.161
Panel B. Difference-in-differences in reverse[b]			
$\text{Post}_{tm} \times \text{Treated}_m$	0.005	0.012	0.019**
	(0.003)	(0.005)	(0.004)
N	51,152	51,152	51,152
mean(y_{imt})	0.102	0.076	0.172
Panel C. Only the four most remote control cities[c]			
$\text{Post}_{tm} \times \text{Treated}_m$	0.006	0.019	0.023*
	(0.004)	(0.011)	(0.008)
N	13,001	13,001	13,001
mean(y_{imt})	0.097	0.07	0.161
Panel D. Controls for insurance product characteristics[d]			
$\text{Post}_{tm} \times \text{Treated}_m$	0.004	0.021*	0.026***
	(0.005)	(0.007)	(0.005)
N	29,332	29,332	29,332
mean(y_{imt})	0.1	0.066	0.16
Panel E. Excluding illnesses which began within one year prior to the entries[e]			
$\text{Post}_{tm} \times \text{Treated}_m$	-0.005	0.025**	0.018
	(0.009)	(0.007)	(0.013)
N	27,086	27,086	27,086
mean(y_{imt})	0.1	0.066	0.16
City and time FEs	✓	✓	✓
Age and sex	✓	✓	✓
Accident/illness FEs	✓	✓	✓

Notes: t-test level of significance: *$<$ 0.05, **$<$ 0.01, ***$<$ 0.001. Standard errors in parentheses. Outcomes: indicator equalling 1 whenever the sum of imaging reimbursements > 0. Standard errors are clustered at the city level. N of clusters in panels B, D and E = 12. N of clusters in panels A and C = 6.

[a] Includes only four (out of ten) largest control cities by population.

[b] The capital region as the always-treated control group.

[c] Includes only four (out of ten) control cities which are the most remote. I retrieved Google Maps driving times from each control city to both treatment cities by car, and used the smaller of the two values as the measure of remoteness (i.e. the driving time to the nearest treatment city).

[d] Added (i) control for the level of cost-sharing and (ii) insurance product fixed effects.

[e] Excluding observations for which $\text{Treated}_m = 1$ and the time (quarters) to treatment equals -1, -2, -3, or -4.

Table A6 Summary statistics: Difference-in-difference in reverse

	Mean	SD	Min	Max	N
Panel A. Accidents					
Radiography or ultrasound (0/1)	0.14	0.35	0	1	14,177
MRI or CT (0/1)	0.26	0.44	0	1	14,177
Any imaging (0/1)	0.38	0.49	0	1	14,177
Panel B. Illnesses					
Radiography or ultrasound (0/1)	0.10	0.30	0	1	51,152
MRI or CT (0/1)	0.08	0.26	0	1	51,152
Any imaging (0/1)	0.17	0.38	0	1	51,152

Notes: Includes accident-/illness-level observations in 2016–2018. Outcomes are based on claims within one year from the accident or the onset of illness.

Table A7 Robustness tests: Alternative outcomes

	Both radiography/ultrasound and MRI/CT (0/1)[a] (1)	Reimbursed medicine expenditures (€)[b] (2)
Panel A. Accidents		
$\text{Post}_{tm} \times \text{Treated}_m$	-0.015	-1.273
	(0.010)	(1.786)
N	8,496	8,496
mean(y_{imt})	0.136	15
Panel B. Illnesses		
$\text{Post}_{tm} \times \text{Treated}_m$	-0.001	1.043
	(0.002)	(1.700)
N	29,332	29,332
mean(y_{imt})	0.1	35
City and time FEs	✓	✓
Age and sex	✓	✓
Accident/illness FEs	✓	✓

Notes: t-test level of significance: *< 0.05, **< 0.01, ***< 0.001. Standard errors in parentheses. Standard errors are clustered at the city level ($N = 12$).

[a] Indicator equalling one if the sum of radiography/ultrasound reimbursements was greater than zero and the sum of MRI/CT reimbursements was greater than zero.

[b] Sum of medicine reimbursements in euros.

Appendix G. Additional figures

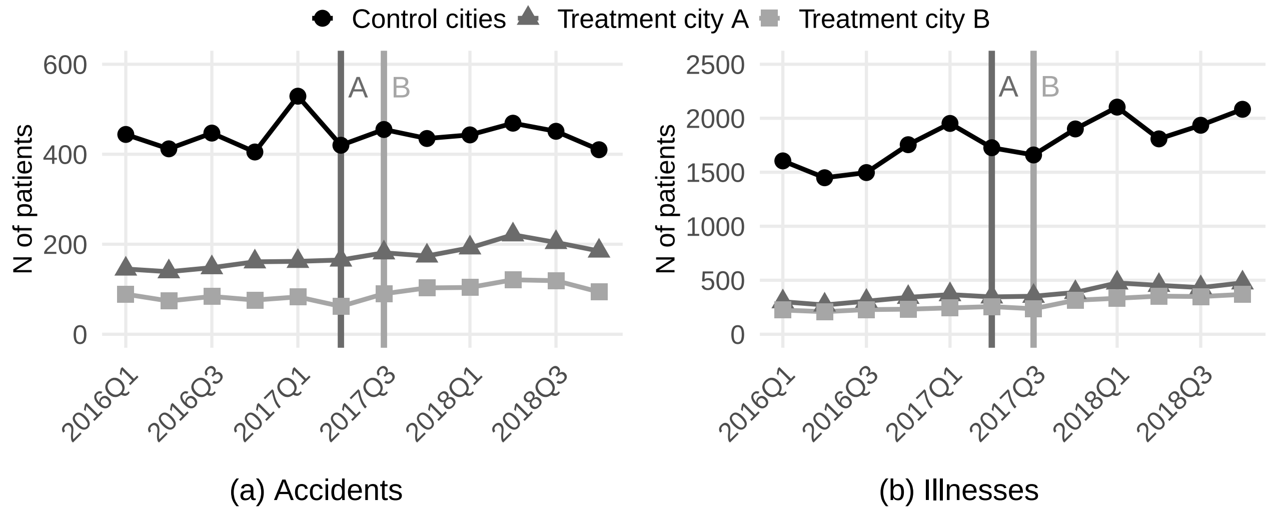

Fig. A1 Number of observations. *Notes:* Includes accident-/illness-level observations in 2016–2018. Control group includes patients in all ten control cities. Vertical lines indicate market entries of the study company's clinics in treatment city A (5/2017) and treatment city B (8/2017).

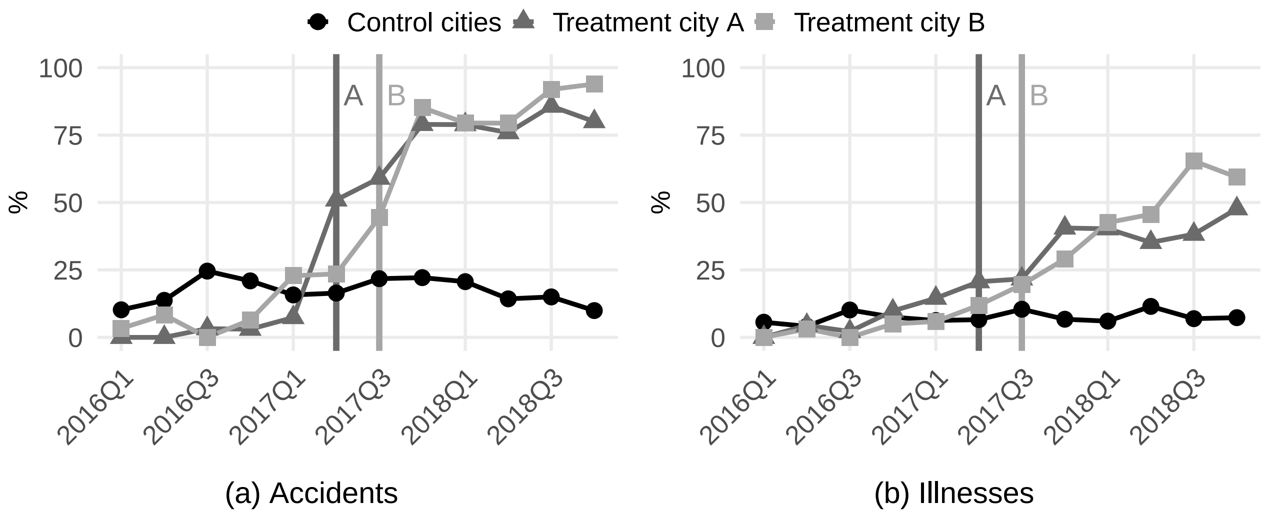

Fig. A2 Share of policyholders' *imaged* accidents and illnesses that were treated in the study company's clinics. *Notes:* The shares were calculated by dividing the number of policyholders' accidents/illnesses reimbursed for imaging in the study company's clinics by the number of all policyholders' accidents/illnesses reimbursed for imaging. The control group includes patients in all ten control cities. Vertical lines indicate market entries of the study company's clinics in treatment city A (5/2017) and treatment city B (8/2017).

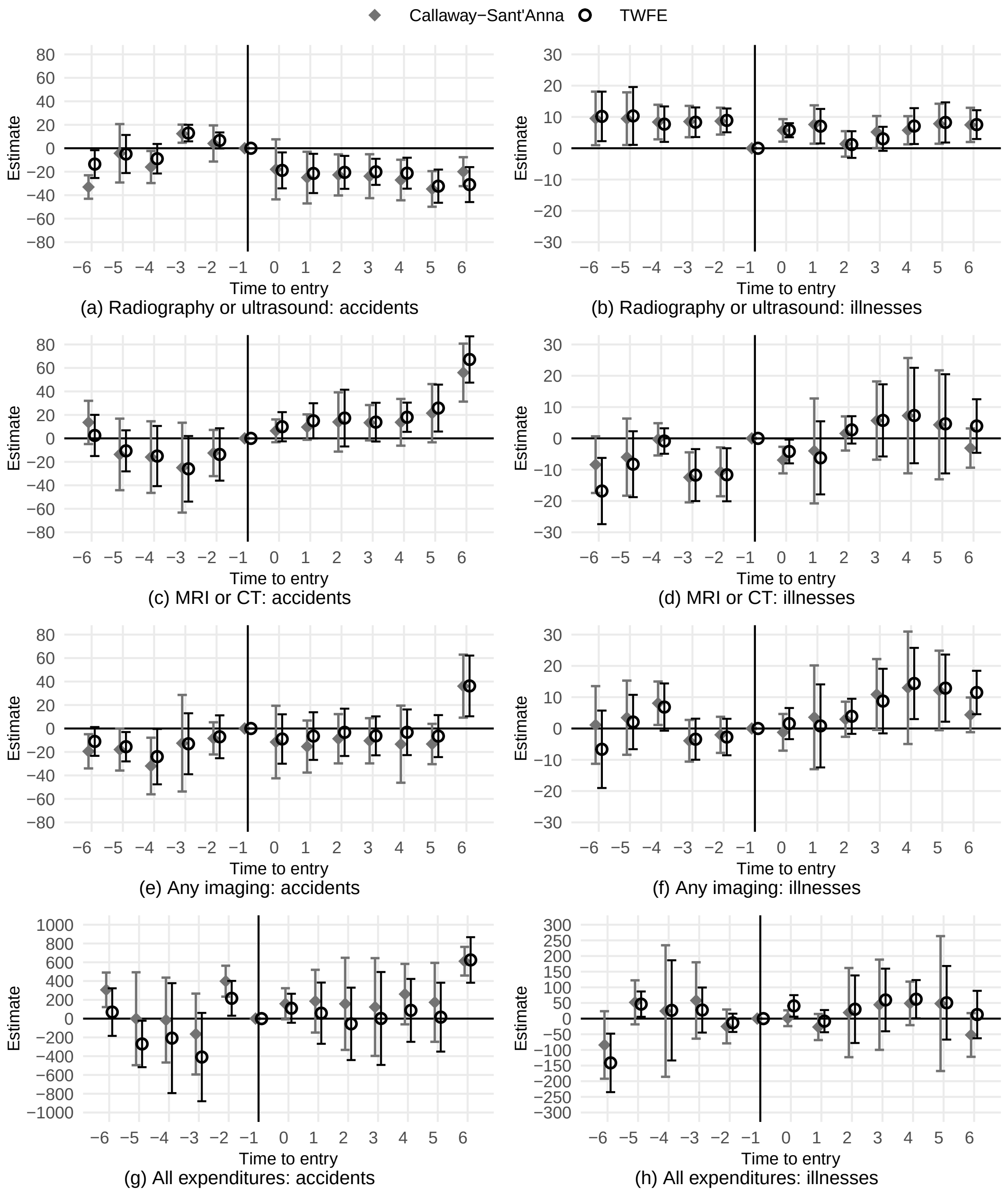

Fig. A3 Monetary outcomes. *Notes:* 95% confidence interval. All outcomes are monetary sums of reimbursements in euros. Estimated using equation (1). Standard errors are clustered at the city level (N of clusters = 12).

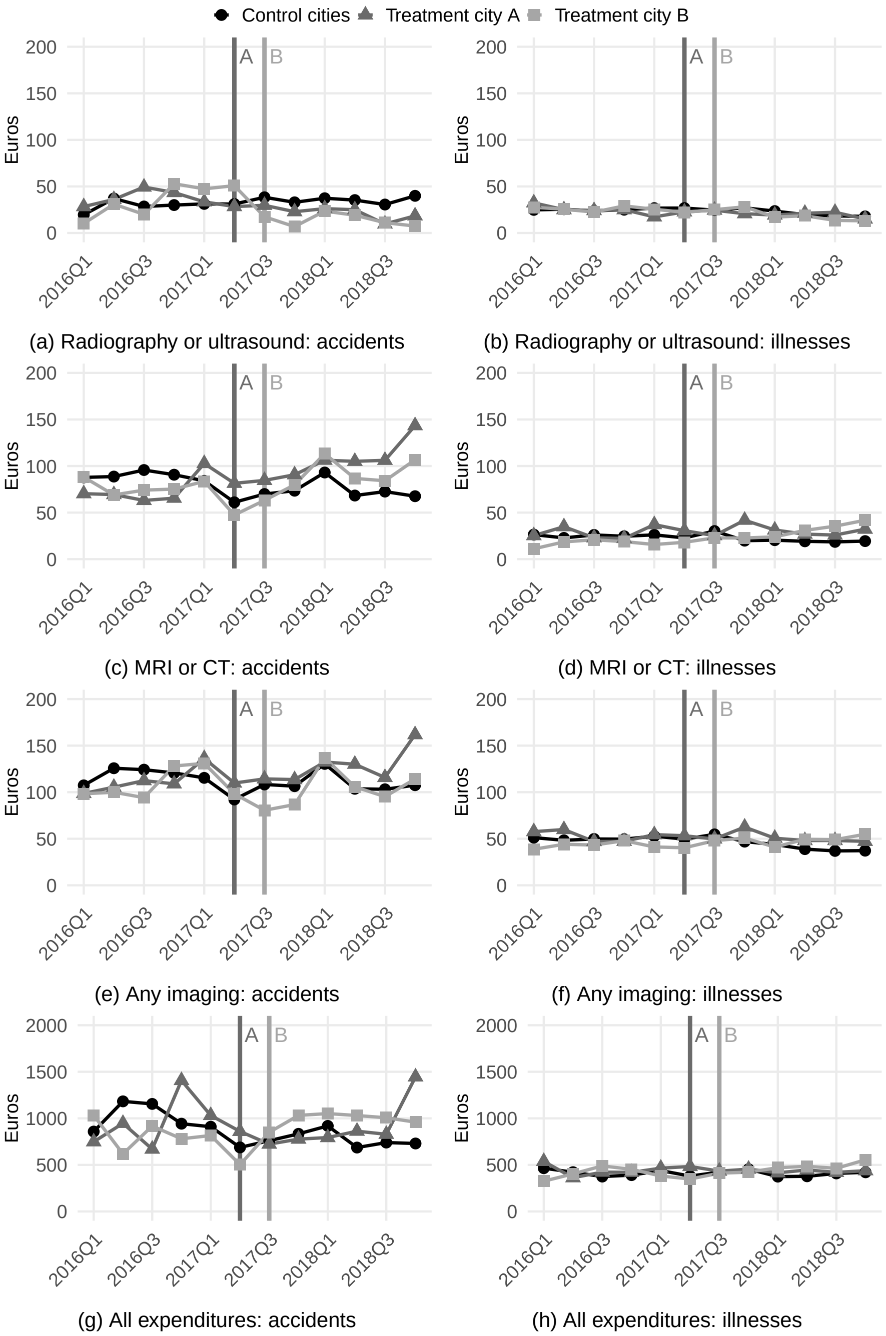

(a) Radiography or ultrasound: accidents
(b) Radiography or ultrasound: illnesses
(c) MRI or CT: accidents
(d) MRI or CT: illnesses
(e) Any imaging: accidents
(f) Any imaging: illnesses
(g) All expenditures: accidents
(h) All expenditures: illnesses

Fig. A4 Outcome means for monetary outcomes. *Notes:* Based on accident-/illness-level data. The means were calculated by dividing the sums of policyholders' reimbursements by the number of policyholders' reimbursed accidents/illnesses. Vertical lines indicate market entries of the study company's clinics in treatment city A (5/2017) and treatment city B (8/2017).

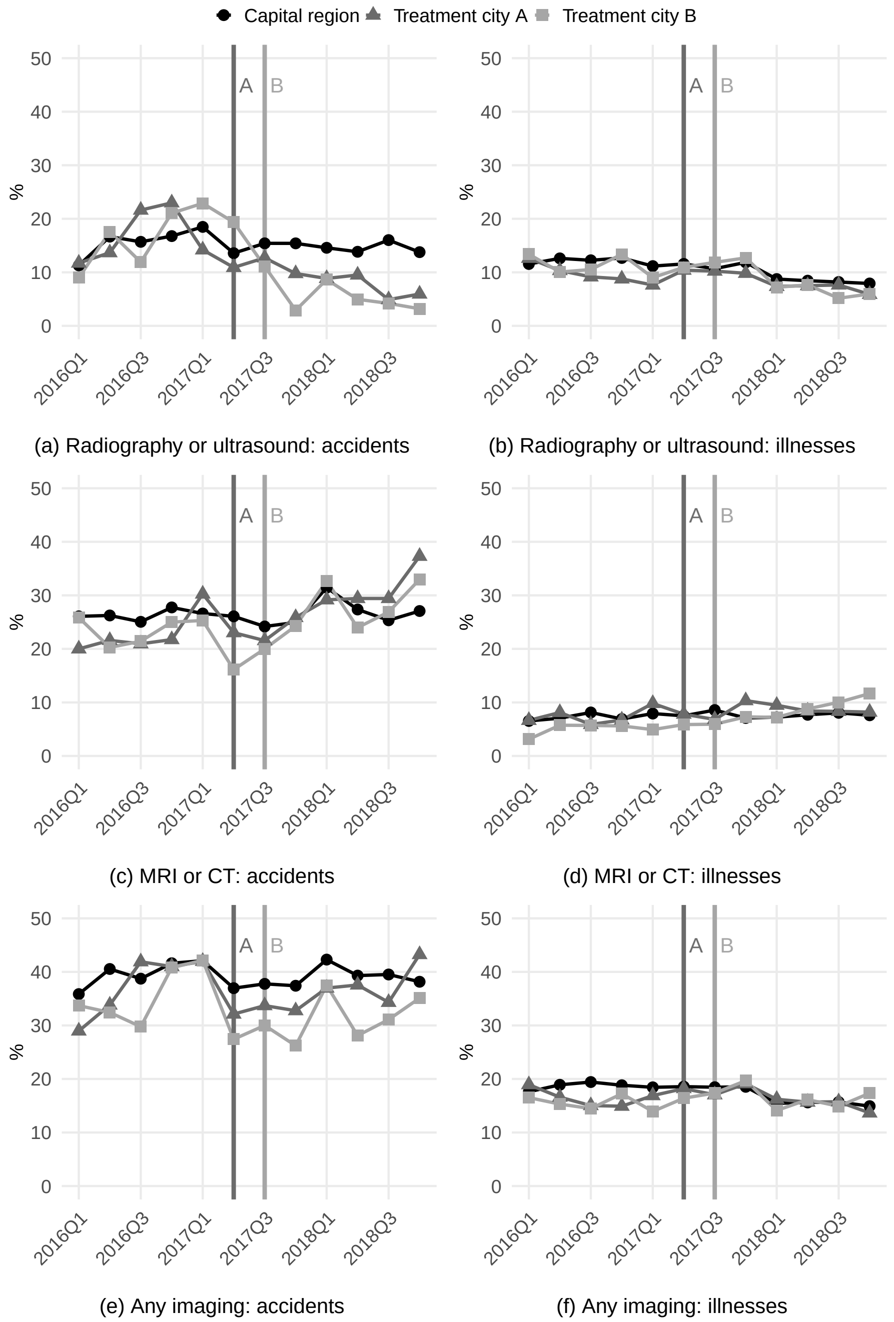

Fig. A5 Outcome means for market entry cities and the always-treated capital region. *Notes:* Based on accident-/illness-level data. The means were calculated by dividing the number of policyholders' accidents/illnesses that were reimbursed for imaging by the total number of policyholders' reimbursed accidents/illnesses. Vertical lines indicate market entries of study company's clinics in treatment city A (5/2017) and treatment city B (8/2017).

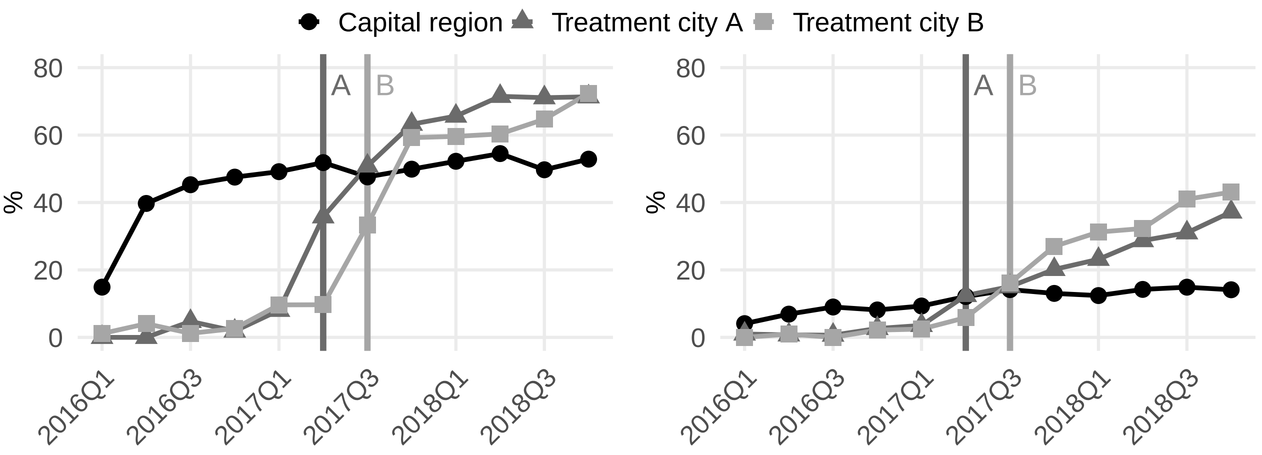

(a) Accidents

(a) Illnesses

Fig. A6 Share of policyholders' accidents that were treated in the study company's clinics: treatment cities vs. always-treated capital region. *Notes:* The means were calculated by dividing the number of policyholders' accidents/illnesses that received care in the study company's clinics by the total number of policyholders' reimbursed accidents/illnesses. The capital region includes three cities. Vertical lines indicate market entries of the study company's clinics in treatment city A (5/2017) and treatment city B (8/2017).

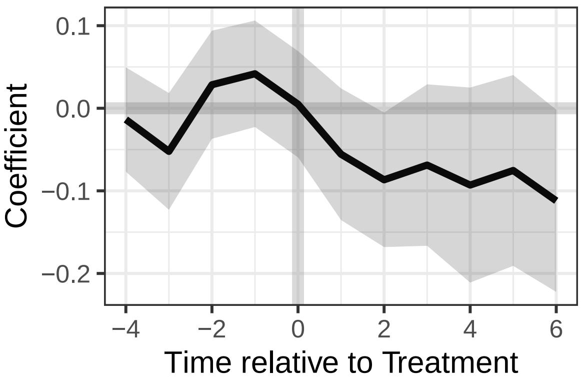

(a) Radiography or ultrasound: accidents

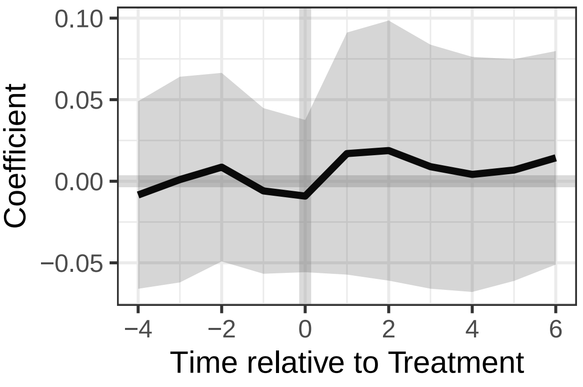

(b) Radiography or ultrasound: illnesses

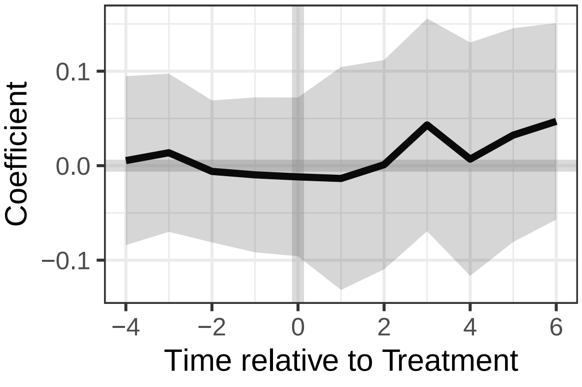

(c) MRI or CT: accidents

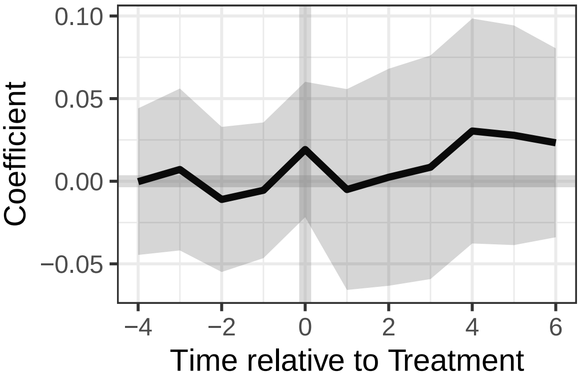

(d) MRI or CT: illnesses

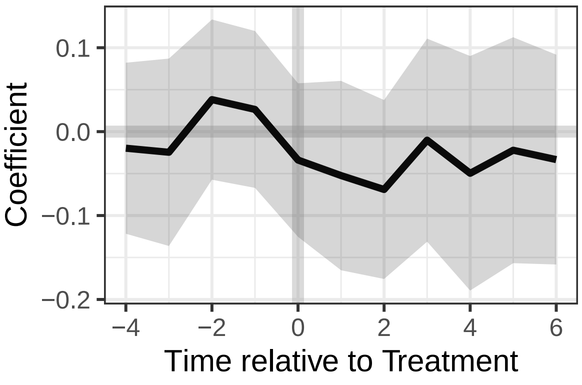

(e) Any imaging: accidents

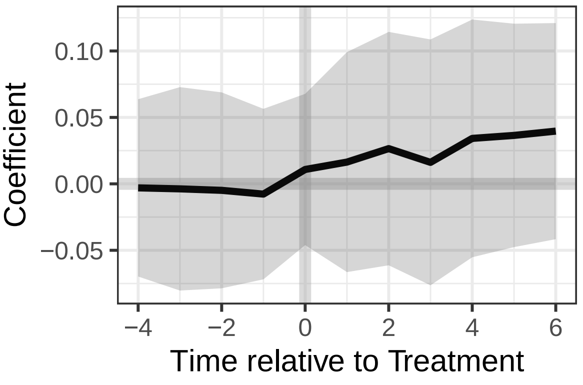

(f) Any imaging: illnesses

Fig. A7 Generalised synthetic control method (GSCM). *Notes:* 95% confidence interval. All outcomes are indicators equalling 1 whenever the sum of imaging reimbursements is > 0. Estimated using the generalised synthetic control method and the same control variables as in equation (1).
